# Metal–organic framework based mixed matrix membranes: a solution for highly efficient CO_2_ capture?†

**DOI:** 10.1039/c4cs00437j

**Published:** 2015-02-18

**Authors:** Beatriz Seoane, Joaquin Coronas, Ignacio Gascon, Miren Etxeberria Benavides, Oğuz Karvan, Jürgen Caro, Freek Kapteijn, Jorge Gascon

**Affiliations:** a Catalysis Engineering, Chemical Engineering Department, Delft University of Technology Julianalaan 131 2628 BL Delft The Netherlands j.gascon@tudelft.nl; b Chemical and Environmental Engineering Department and Instituto de Nanociencia de Aragón (INA), Universidad de Zaragoza 50018 Zaragoza Spain; c Physical Chemistry Department and Instituto de Nanociencia de Aragón (INA), Universidad de Zaragoza 50018 Zaragoza Spain; d Tecnalia, Energy and Environmental Division, Parque Tecnológico de San Sebastián Mikeletegi Pasealekua 2. E-20009 Donostia-San Sebastian Spain; e Institut für Physikalische Chemie und Elektrochemie, Leibniz Universität Hannover Callinstr. 22 D-30167 Hannover

## Abstract

The field of metal–organic framework based mixed matrix membranes (M^4^s) is critically reviewed, with special emphasis on their application in CO_2_ capture during energy generation. After introducing the most relevant parameters affecting membrane performance, we define targets in terms of selectivity and productivity based on existing literature on process design for pre- and post-combustion CO_2_ capture. Subsequently, the state of the art in M^4^s is reviewed against these targets. Because final application of these membranes will only be possible if thin separation layers can be produced, the latest advances in the manufacture of M^4^ hollow fibers are discussed. Finally, the recent efforts in understanding the separation performance of these complex composite materials and future research directions are outlined.

## Abstract 2

The field of metal–organic framework based mixed matrix membranes (M^4^s) is critically reviewed, with special emphasis on their application in CO_2_ capture during energy generation.

## Graphical abstract

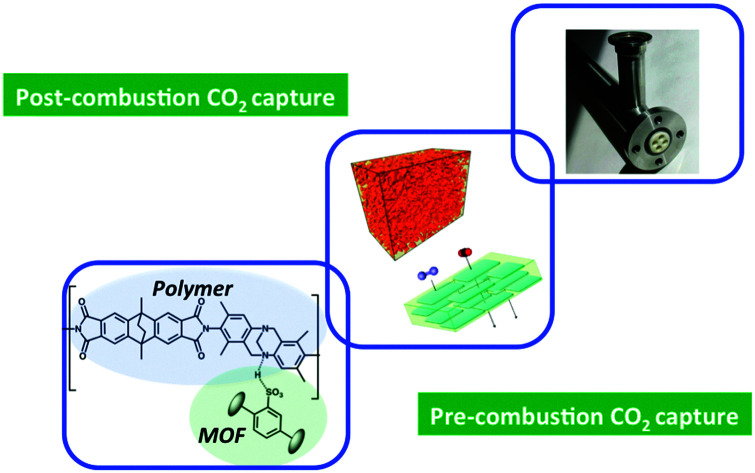

## A Introduction – setting the scene

The urgent need for strategies to reduce global atmospheric concentrations of greenhouse gases has prompted international action from governments and industries, and a number of collaborative programs have been established including the European Strategic Energy Technology Plan (SET-Plan), the European Technology Platform for Zero Emission Fossil Fuel Power Plants (ZEP), the Intergovernmental Panel on Climate Change (IPCC), the United Nations Framework Commission on Climate Change, and the Global Climate Change Initiative.^1–4^ In addition to the continuous development of non CO_2_ emitting generation of energy from wind, solar or hydro and geothermal sources, the capture and sequestration of carbon dioxide, the predominant greenhouse gas, is a central strategy in these initiatives, as it offers the opportunity to meet increasing demands for fossil fuel energy on the short- to medium-term, whilst reducing the associated greenhouse gas emissions.^5^ In this spirit the EU, through the SET-Plan and the CCS Technology Roadmap, has agreed to enable the cost competitive deployment of CCS after 2020 and to further develop the technologies to allow application in all carbon intensive industrial sectors, with an objective of 90% CO_2_ capture with less than 8 percentage point efficiency losses.^2,3,6^

**Fig. 1:**
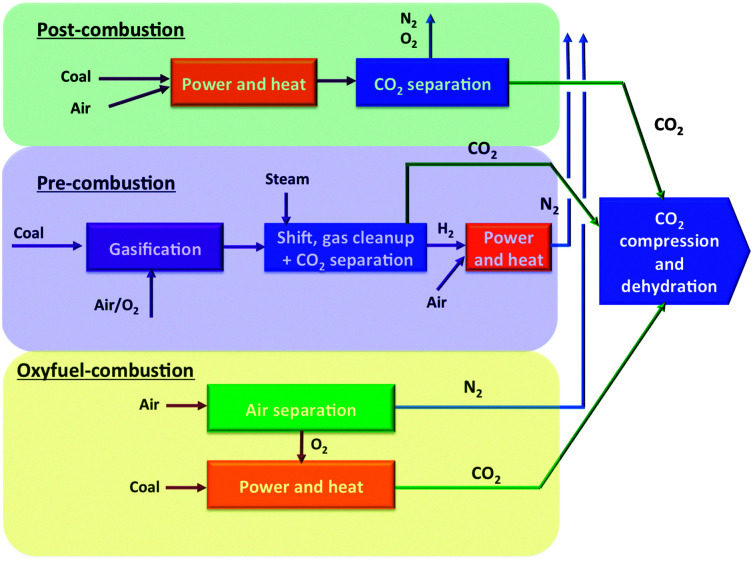
Technical options for CO_2_ capture from coal-power plants.^1^

Broadly, three lines of capturing technologies exist to reduce CO_2_ emissions in combustion processes: post-combustion, pre-combustion, and oxyfuel combustion (Fig. 1).

Post-combustion CO_2_ capture comprises of capturing CO_2_ from the flue gases produced after fossil fuels or other carbonaceous materials (such as coal or biomass) are burned. Combustion-based power plants provide most of the world's electricity today. In modern natural gas and coal-fired power plants, the combustible is mixed with air and burned. The heat released by combustion generates steam, which drives a turbine-generator. The hot combustion gases exiting the boiler consist mainly of nitrogen (from air) plus lower concentrations of water vapour and CO_2_, with the concentration of the latter depending on the combustible used. Additional products formed during coal combustion from impurities in coal include sulphur dioxide, nitrogen oxides and particulate matter (fly ash). These regulated air pollutants, as well as other trace species such as mercury, must be removed to meet applicable emission standards. In some cases, additional removal of pollutants (especially SO_2_) is required to provide a sufficiently clean gas stream for subsequent CO_2_ capture. The absence of impurities in natural gas results in a clean flue gas stream, so that no additional clean-up is needed for effective CO_2_ capture.^7^

With current technology, the most effective method of CO_2_ capture from flue gases is chemical absorption in an aqueous solution of an amine based organic, such as mono- or diethanolamine (MEA, DEA). In the absorber, the flue gas is countercurrently “scrubbed” with an amine solution, typically capturing 85 to 90 percent of the CO_2_. The CO_2_-laden solvent is then pumped to a second vessel (stripper), where heat is supplied in the form of steam to release the CO_2_. The resulting stream of concentrated CO_2_ is then compressed and piped to a storage site, while the depleted solvent is recycled back to the absorber. The regeneration requires considerable energy, as not only the captured CO_2_ has to be released at higher temperatures, also the evaporation losses of water are considerable.^1^

To remove carbon from fuel prior to combustion it must first be converted into a form amenable to capture. For a coal-fuelled plant this is accomplished by reacting coal with steam and oxygen at high temperature and pressure, a process called partial oxidation, or gasification. The result is a gaseous fuel consisting mainly of carbon monoxide and hydrogen mixture known as synthesis gas (syngas), which can be burned to generate electricity in a combined cycle power plant. This approach is known as integrated gasification combined cycle (IGCC) power generation. After particulate impurities are removed from the syngas, a two-stage shift reactor converts the carbon monoxide to CO_2_*via* a reaction with steam (H_2_O). The result is a mixture of CO_2_ and hydrogen (and water). A solvent, such as the widely used commercial Selexol® (which employs a glycol-based solvent) and Rectisol® (using refrigerated methanol), then captures the CO_2_, leaving a stream of nearly-pure hydrogen that is burned in a combined cycle power plant to generate electricity.^8^ Although the fuel conversion steps of an IGCC plant are more elaborate and costly than traditional coal combustion plants, CO_2_ separation is much easier and cheaper because of the high operating pressure and high CO_2_ concentration of this design. Thus rather than requiring a chemical reaction to capture CO_2_ (as with amine systems in post-combustion capture), the mechanism employed in pre-combustion capture involves physical absorption into a solvent (although pressures above ∼20 bar are required), followed by release of the CO_2_ when the pressure is reduced, typically in several stages. Nonetheless, there is still a significant energy penalty associated with CO_2_ capture due to the need for a shift reactor and other processes. In oxyfuel processes pure oxygen is used for the combustion, resulting in a flue gas containing mainly water vapour and carbon dioxide. Condensation of the water results in a nearly pure carbon dioxide stream. The major energy penalty here is the production of pure oxygen by air separation.

In general, the higher the power plant efficiency, the smaller the energy penalty and associated impact for CO_2_ separation. For this reason, replacing or repowering an old, inefficient plant by a new, more efficient unit with CO_2_ capture can still yield a net efficiency gain that decreases all plant emissions and resource consumption. Thus, the net impact of the CO_2_ capture energy penalty must be assessed in the context of a particular situation or strategy for reducing CO_2_ emissions and developing sustainable processes.^9^

The energy requirements of current CO_2_ capture systems are roughly ten to a hundred times greater than those of other environmental control systems (*e.g.* de-NOx, SOx capture, fly ash removal) employed at a modern electric power plant. This energy “penalty” lowers the overall (net) plant efficiency globally by 20–30% and significantly increases the net costs of CO_2_ capture, as indicated in Table 1.

**Table 1:** Representative values of power plant efficiency and CCS energy penalty. All efficiency values are based on the higher heating value (HHV) of fuel^7,10^

Power plant and capture system type	Net plant efficiency (%) w/o CCS	Net plant efficiency (%) with CCS	CCS energy penalty	
			Additional energy input (%) per net kW h output^a^	Reduction in net kW h output (%) for a fixed energy input
Existing subcritical PC,^b^ post-combustion capture	33	23	43	30
New supercritical PC, post-combustion capture	40	31	29	23
New supercritical PC, oxy-combustion capture	40	32	25	20
New IGCC (bituminous), pre-combustion capture	40	33	21	18
New Natural Gas comb. cycle, pre-combustion capture	50	43	16	14

a This is the definition of the incremental primary energy needed to supply one unit of electric power (*e.g.*, 1 kW h) to the grid.

b PC stands for pulverized coal.

A recent analysis has shown that the thermodynamic minimum energy demand for capturing 90% of the CO_2_ from the flue gas of a typical coal-fired power plant is approximately 3.5% (assuming a flue gas containing 12–15% CO_2_ at 40 °C).^11^ By comparison with data presented in Table 1, column 5, it is clear that current technology is far from ideal. In addition, although absorber–stripper units represent a proven, well-accepted technology in the gas processing industry, the high-pressure absorber tower in particular is an expensive, large, thick-walled, heavy vessel. The need to heat and cool the recirculating fluids requires careful, well-monitored, expensive operating procedures.^12,13^ Furthermore, the degradation of the amine absorbent leads to corrosive mixtures. Although the use of inhibitors reduces degradation (*e.g.* FLUOR's Econamine FGplus technology^14^), the need for regular maintenance hinders the use of amine absorber–strippers in remote locations and in small electricity plants.^15^ On the other hand, the use of amines and solvents is environmentally unfriendly due to the contamination of the gas with solvent vapours and the likely degradation of amines due to the high temperature treatments required to regenerate the absorbent. To prevent this, task specific ionic liquids, which exhibit extremely low partial pressures up to 300 °C, have been designed as solvents or active absorbents. However, the frequently used ionic liquids with phosphine anions have the tendency to decompose *via* Beckman rearrangement at moderate temperatures^16^ which limits their use.

**Fig. 2:**
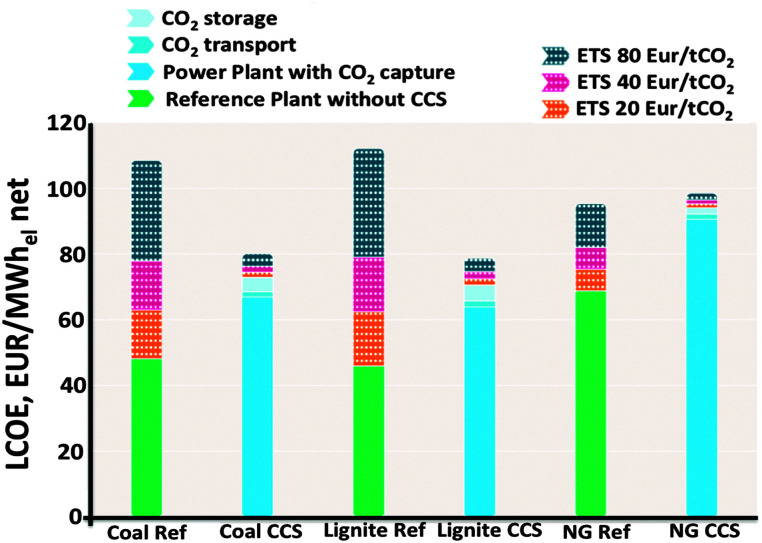
The levelized cost of energy (LCOE) of integrated CCS projects (blue bars) compared to the reference plants without CCS (green bars).^1^

Fig. 2 displays the Levelized Cost Of Energy (LCOE) of new power plants with and without CCS, considering different Emissions Trading System (EU ETS) values as reported in recent studies based on current commercial post-combustion and pre-combustion capture processes.^1^

**Fig. 3:**
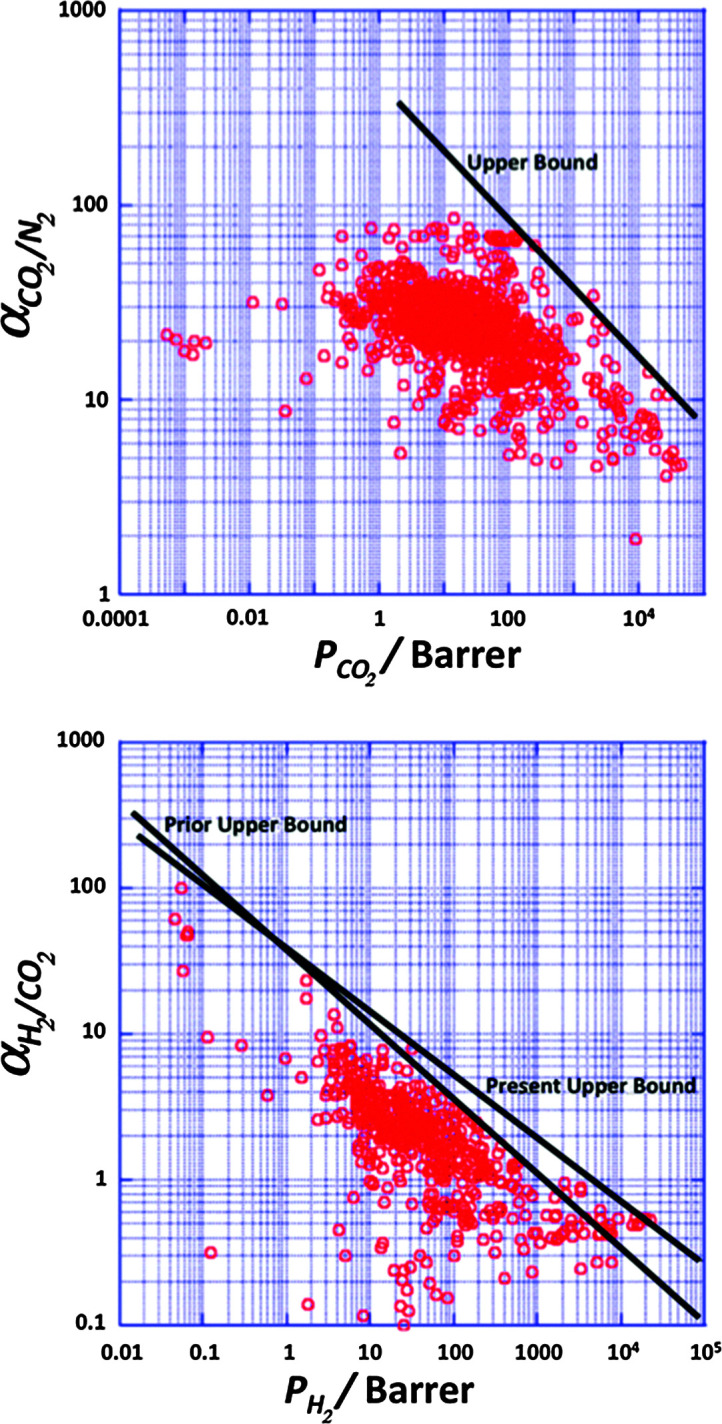
Robeson plots for the separation of CO_2_ from N_2_ (top) and H_2_ from CO_2_ (bottom). This plot shows the selectivity obtained from the ratio of pure-gas permeabilities plotted against permeability of one component for different polymeric membranes. No commercial polymeric membranes currently operate above the upper bound.^23^

The LCOE (in € per MW h) is shown for power plants burning bituminous coal, lignite or natural gas. The LCOE includes the costs of CCS, from which 80 to 90 percent is for capture (including compression), the rest for transport and storage.^11^Fig. 2 can be used to calculate the cost per MW h of electricity produced: for a coal reference plant 45 € per MW h without CCS *vs.* 70 € per MW h using CCS. This cost difference is equivalent to the “carbon price”, for new supercritical coal plants this is currently about €24–35/ton CO_2_ (one should note that the amount of tons of CO_2_ released per MW h of electricity generated depend on the efficiency of the plant on the nature of the coal).

These economic, energetic, operational and environmental evaluations underscore the immense opportunities and incentives that exist for improved CO_2_ capture processes and materials.

Alternative processes, still in different stages of development, comprise chemical looping combustion (CLC) using metal oxides, carbon capture during water gas shift (clay type materials), and adsorption–regeneration processes using solid adsorbents (zeolites, activated carbons, metal–organic frameworks). These are all cyclic uptake–regeneration processes based on solid materials, that are either recirculated from one reactor system to another, or used in fixed beds in swing operation.^17–19^ These processes operate at quite different temperature levels, with the adsorption–desorption processes at the lowest since these are mainly based on (exothermal) physical adsorption.

Gas separation membranes offer a number of benefits over other gas separation technologies.^20^ Conventional technologies such as the cryogenic distillation, adsorption, condensation and amine absorption require a gas–liquid phase change. This phase change adds a significant energy cost to the separation cost. Membrane gas separation, on the other hand, does not require a phase change. In addition, gas separation membrane units are smaller than other types of plants, such as amine stripping plants, and therefore have relatively small footprints. The lack of mechanical complexity in membrane systems is another advantage. Membrane devices for gas or vapour separation usually operate under continuous, steady-state conditions. The feed stream passes along one side of the membrane. The non-permeating molecules that are retained at the feed-stream side exit the membrane as the retentate stream. A pressure difference across the membrane drives the permeation process. The mechanism of permeation (sorption of molecules and diffusion) depends on the membrane material. In case of membranes with well-defined pores (*i.e.* zeolites, metal–organic frameworks, carbon molecular sieves (CMS)) adsorption, diffusion and eventually molecular sieving dominate membrane performance, whereas in case of polymeric membranes permeation takes place mostly through a solution–diffusion mechanism.

In 1980, Permea (now a division of Air Products) launched its hydrogen-separating Prism membrane.^21^ This was the first large industrial application of gas separation membranes. Since then, membrane-based gas separation has grown almost exponentially.^22,23^ Membranes were known to have the potential to separate important gas mixtures long before 1980, but the technology to fabricate economically high-performance membranes and modules was lacking. The development of high-flux anisotropic membranes and large surface area membrane modules for reverse osmosis applications occurred in the late 1960s and early 1970s. Permea then adapted this technology to membrane gas separation.^24^ Its polysulfone hollow-fiber membrane was an immediate success, particularly for the separation and recovery of hydrogen from the purge gas streams of ammonia plants. Within a few years, Permea systems were installed in many such plants. This success encouraged other companies to advance their own technologies.

The first membrane systems (anisotropic cellulose acetate) to separate carbon dioxide from natural gas were introduced in the mid 1980s by Cynara (now part of Natco), Grace Membrane Systems Separex (now part of UOP), and GMS (now part of Kvaerner).^25^ In the last decade, cellulose acetate has begun to be challenged by newer membranes, such as polyimide (Air Liquide) and perfluoropolymer membranes (ABB/MTR).^21^ At about the same time, Generon (now part of MG) introduced a membrane system based on poly(4-methyl-1-pentene) (TPX) to separate nitrogen from air. These membranes were only competitive in a few niche areas requiring 95% nitrogen, but by 1990, Generon, Praxair, and Medal all had produced custom polymers with higher oxygen selectivities.^25^ This application has grown to represent about one-third of the new nitrogen production capacity; to date more than 10 000 nitrogen systems have been installed worldwide.^22^ Finally, membranes are also being used for a variety of small but growing applications, such as the dehydration of compressed air and the separation of hydrocarbons from nitrogen or air.^25^

As observed above, to date only polymeric membranes have found their way towards large-scale industrial implementation in gas separation. This is to a large extent due to their easy processing and mechanical strength.^26^ However, a poor resistance to contaminants, low chemical and thermal stability and a limit in the trade-off between permeability and selectivity, the so called Robeson upper bound limit,^22,23^ are among their main disadvantages.

In parallel to the development of polymeric membrane materials, much research effort has been devoted to develop pure inorganic membranes, among others by several of our authors. Inorganic membranes refer to membranes made of materials such as ceramics,^27^ carbon,^28^ zeolite,^29^ various oxides (alumina, titania, zirconia),^30^ metal–organic frameworks,^31^ and metals such as palladium, silver and their alloys.^32^ Inorganic membranes can be classified into two major categories based on their structure: porous inorganic membranes and dense (non-porous) inorganic membranes. Microporous inorganic membranes include both amorphous and crystalline membranes. Although inorganic membranes offer unique properties for gas separation (*i.e.* excellent thermal and chemical stability, good erosion resistance and high gas flux and selectivity), certain aspects still require further attention such as mechanical resistance, reproducibility, long term stability, scaling up and, more importantly, fabrication costs. Other types of hybrid membranes such as organosilica based^33,34^ share the same fabrication prize issue.

The cost of inorganic membranes is dominated by that of the support on which the selective layer is deposited, with Pd membranes as exception.^31,35^ Only zeolite A membranes are deployed commercially for alcohol dehydration by vapour permeation.

In order to overcome the limitations of both polymeric and inorganic membranes, the so-called Mixed Matrix Membranes (MMMs, consisting of a blend of filler particles in a polymeric matrix) have been identified to provide a solution to go beyond the upper-bound trade-off limit of the polymeric membranes as well as the inherent obstacles of brittleness and lack of reproducibility associated with inorganic membranes. MMMs potentially combine the advantages in separation performances of both inorganic and polymeric membranes and overcome their drawbacks, although it introduces the issue of compatibility between the constituents. A good adhesion is essential to avoid non-selective voids in such membranes.

Indeed, during the last few decades, several solutions have been proposed to boost the performance of polymeric membranes. Various polymers have been modified with inorganic fillers such as zeolites, mesoporous silicas, activated carbons, carbon nanotubes and even non-porous solids to produce Mixed Matrix Membranes (MMMs).^36–40^ A mixed matrix membrane is a composite of filler particles in a polymeric matrix. As it will unveiled in this review, both polymer as well as filler properties affect MMM morphology and separation performance.

Recent advances have shifted towards the addition of new fillers, namely carbon nanotubes, layered silicates (sometimes after delamination) and MOFs as potential fillers in the polymer matrix.^41,42^ MOFs are among the most sophisticated nanostructured materials.^43^ In addition to a high surface area and pore volume, their chemical nature can be fine-tuned by selecting the appropriate building blocks and/or by post-synthetic modification, thus leading to tailored porous materials with great promise for the selective adsorption of strategic gases. More importantly, the porosity of MOFs is, in general, much higher than that of their inorganic counterpart, zeolites, justifying the designation ‘framework’ and challenging the scientific community to make an effective use of such empty space. In addition to the facile functionalization, many MOFs are known to undergo structural changes upon adsorption of different molecules (‘breathing’),^44^ facilitating the design of, for instance, dynamic composites.^45–47^ When it comes to MMMs, the use of MOFs as fillers might result in a breakthrough in the MMM field, since compatibility issues can eventually be overcome by optimizing the MOF linker–polymer interactions.^46^ Since the first report in 2004,^48^ research into M^4^s has experienced an unprecedented explosion. Certainly, as highlighted in recent reviews,^49–52^ MOF based mixed matrix membranes (M^4^s) have the potential to overcome current challenges in membrane separation, both in terms of membrane synthesis and performance. Because of these reasons, we believe that it is now the right moment to critically evaluate the recent advances in the field. In this review, after introducing the most relevant parameters affecting membrane performance, we will define targets in terms of selectivity and productivity based on existing literature on process design for pre- and post-combustion CO_2_ capture. Subsequently, the state of the art in M^4^s is reviewed against the previously defined targets. Because final application of these membranes will only be possible if thin separating layers can be produced, we will then review the latest advances in the manufacture of M^4^s hollow fibers. Last but not least, the recent efforts in understanding the separation performance of these complex composite materials will be discussed. This article is finally wrapped up with our personal opinion and possible future directions in the development of new generations of M^4^s.

## B Describing transport in mixed matrix membranes

The lab-scale manufacture of M^4^s is similar to the one applied for the synthesis of other MMMs. In the general procedure, the first step is the dispersion of the filler in the solvent in an ultrasonic bath. Polymer is then added, usually maintaining a ratio 90/10 wt% solvent/filler–polymer mixture. The whole mixture is stirred overnight. Before the casting, different intervals of sonication and stirring take place to ensure a well dispersion, provided that sonication does not result in deterioration of the polymer (something very important when high flux polymers such as PIM-1 are used in the manufacture of the composite). Subsequently, the membranes are cast on a flat surface, either Petri-type dishes or Doctor Blade system, and then left overnight for evaporation of solvent at room temperature. Once dried, the films are placed in a vacuum oven for 24 h at a specific temperature (depending on the polymer glass transition temperature) high enough to remove the remaining solvent.

Permeability and separation factor are the two key parameters generally used to characterize polymeric membranes. Permeability, officially called permeability coefficient, *P*_*i*_, a normalized productivity of a specific gas component by the membrane, is defined (eqn (1)) as the diffusive Flux of gas *i* through the membrane (flow per unit membrane area *A*) normalized by the partial pressure difference of that component across the membrane per unit thickness of the membrane (*l*).1
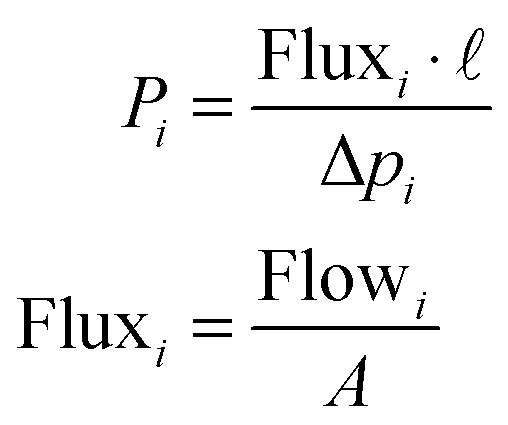
 Permeability values are typically reported in Barrer units (1 Barrer = 1 × 10^−10^ cm^3^(STP) cm cm^−2^ s^−1^ cmHg^−1^ = 3.344 × 10^−16^ mol m m^−2^ Pa^−1^ s^−1^).

However, permeability values can only be given when the thickness of the separating layer is well known, something not possible in case of very thin membranes or advanced membrane configurations such as hollow fibers and asymmetric films. In this case, Permeance (pressure normalized flux) is used, with Gas Permeance Units (GPU) being the most widely applied units in polymer membrane separations: 1 GPU = 10^−6^ cm^3^(STP) cm^−2^ cmHg^−1^ = 0.344 × 10^−10^ mol m^−2^ s^−1^ Pa^−1^.

The separation factor or permselectivity reflects the capability of a membrane to separate one gas from another. If the permeabilities of two individual components are known, the ideal selectivity, *S*_*ij*_ (eqn (2)), is given by the ratio of the two pure gas permeabilities:2
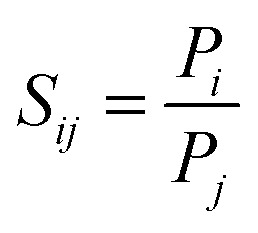
 For permeation of actual *i*–*j* mixtures, the mixed gas selectivity, also called separation factor (*α*_*ij*_), is calculated from composition analysis as the ratio of the mole fractions, *X*, of the components in the permeate stream, and the retentate stream (eqn (3)). In the case where the gases do not interact strongly with each other or with the membrane material, the ideal selectivity is equal to the actual separation factor, but often this is not the case.^53^3
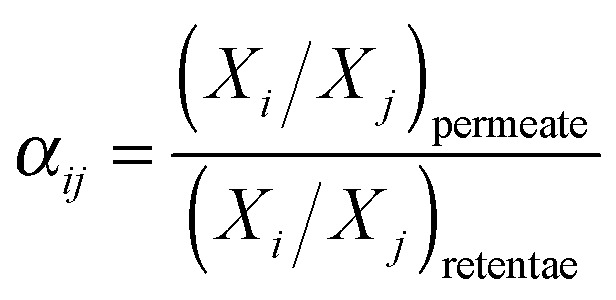
 Gas permeation transport in MMMs is governed by the combination of a solution–diffusion mechanism in the continuous polymer matrix and permselective transport through the dispersed MOF. In case of the latter, two different contributions are expected: (i) adsorbate–surface interactions, concerning chemical and/or physical interaction between the adsorbent and the adsorbate; and (ii) size-exclusion, related to the dimension and shape of the framework pores and of the molecules.^54^

In order to be able to understand composite performance, the development of appropriate models that describe transport is crucial. For an extensive overview on modeling of MMMs we strongly recommend the recent review by Vinh-Thang and Kaliaguine.^55^ In short, during the last few decades different models have been proposed to estimate the permeation performance of MMMs by developing different theoretical expressions depending on MMM morphology. These models capture to different levels of complexity the presence of filler in a continuous polymer matrix, and the effect of voids and rigidified polymer regions. Barrer and Petropoulos were the first proposing a model for the performance of polymer–filler blends.^56^ Their formulation assumes concentration-independent diffusivities and Henry's law for adsorption, and it deals with the inherent two-dimensionality of the situation through the introduction of several unknown correction factors. The reliance on such correction factors is one good reason to seek more satisfying treatments; the restrictive assumption of Henry's law adsorption is another. Cussler has proposed perhaps the most sophisticated model by reducing the three-dimensional diffusion problem to an essentially one-dimensional problem through a series of approximations.^57^ Aside from the limitations of these approximations, Cussler's model employs Fickian diffusivity with a constant diffusivity and an equilibrium condition between phases that requires identical adsorption isotherms in both materials. These are both serious limitations, particularly when trying to describe the performance of a composite containing a zeolite phase. The Maxwell formulation can be also extended to MMMs by combining the flux through the polymer and filler in parallel and series pathways, in a clear analogy to electrical circuits. This model is however only applicable for low filler loadings since it assumes that the streamlines associated with diffusive mass transport around filler particles are not affected by the presence of nearby particles. The Bruggeman model,^58^ which can be considered to be an improved version of the Maxwell model, accounts for these effects and extends the applicability to highly loaded MMMs.^59^ Sheffel and Tsapatsis^60,61^ introduced later a more extended model for diffusive transport in microporous MMMs utilizing the Maxwell–Stefan formulation and different models to account for multi-component mixtures: Henry's law, extended Langmuir model, and ideal adsorbed solution theory (IAST).

Any model attempting to describe diffusion within a membrane containing a microporous filler phase must include a realistic treatment of diffusion in both the porous filler and the continuous phase. Diffusion in the gas phase or in relative large pores (>100 nm (ref. 62)) is dominated by inter-molecular collisions and the flux of component *i* can be described by the Maxwell–Stefan (MS) approach,^63^ in which forces acting on molecules (in diffusional processes the gradient in thermodynamic potential) are balanced by the friction between molecules and, in case of porous materials, with a solid. In the latter case this model was named the ‘Dusty Gas Model’. The often used Fick's law is a simplification of the generalized MS equations for thermodynamically ideal systems.^63^

While Fickian diffusion can be used to describe transport through polymers, in the case of a porous material a correction needs to be made to account for the porosity (*ε*) and tortuosity (*τ*) of the material, leading to an ‘effective’ diffusivity. In this way, molar flux (*N*_*i*_, mol m^−2^ s^−1^) can be defined as:4
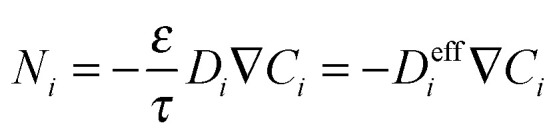
 In porous materials, when the mean free path of a molecule is in the order of or larger than the pore diameter (∼10–100 nm) molecule-wall collisions start to dominate and the diffusivity can be described by the Knudsen diffusion mechanism. A flux in such small pores can be presented as:5
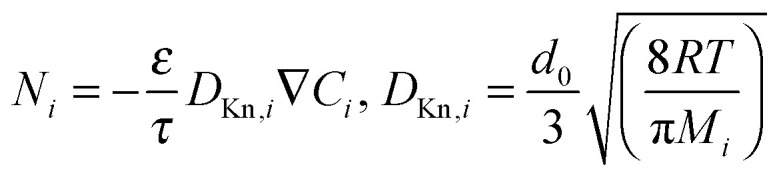
 In the case of zeolites and MOFs, the pores approach molecular dimensions (∼0.3–0.74 nm) and, consequently, mass transport through such pores is determined by the interaction of the molecules with the pore wall. Now molecules are adsorbed, have lost their gaseous nature, and transport is often referred to as surface or zeolitic diffusion.^62^ The flux can now also be represented in a Fickian way; the concentration (*q*_*i*_) represents the adsorbed amount or loading. A common unit for the loading is mol kg^−1^, therefore the adsorbent density (*ρ*) is added to arrive at consistent dimensions. Note that the diffusivity in this case has a different magnitude by this definition (compare eqn (6) and (5)), about a factor of the Henry constant for adsorption.

6 *N*_*i*_ = −*ρD*_*i*_∇*q*_*i*_

The tortuosity and porosity presented in eqn (4) are not specified in eqn (6), these are an inherent property of the diffusivity. Each adsorbent has its own specific pore network with its own tortuosity and porosity. Moreover, the pore network can be 1-, 2- or 3-dimensional with different pore sizes or connectivities in different directions leading to diffusion anisotropy.^64^

The adsorbed phase (*q*_*i*_) in eqn (6) is related to the gas phase fugacity through an adsorption isotherm of which the classical example is the Langmuir isotherm:7
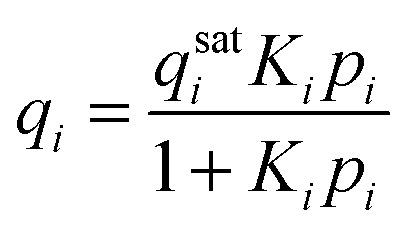
 An important difference between gas phase and adsorbed phase diffusion is the concentration level, being much higher in the case of adsorbed phase diffusion. When the gradient in chemical potential is taken as the fundamental driving force for diffusion^62,63^ a correction needs to be made to eqn (6). Now, a so-called thermodynamic correction factor (*Γ*_*ii*_) is introduced; the diffusivity is referred to as ‘corrected’ or ‘Maxwell–Stefan’ (MS) diffusivity.8
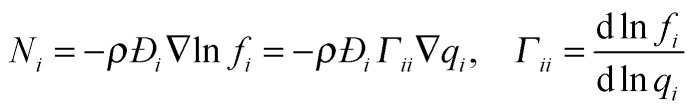
 For a single site Langmuir isotherm the thermodynamic correction factor is given by:9
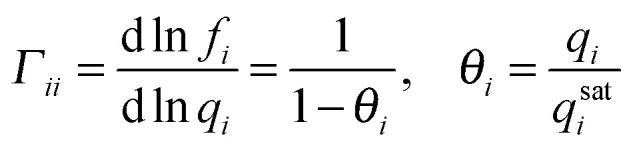
 In the limit of low loading the thermodynamic correction factor approaches 1 and the MS and Fickian diffusivity are equal. Although the MS diffusivity appears to be physically more correct, the Fickian diffusivity remains very important since this diffusivity can be directly assessed in diffusion measurements.

When multicomponent adsorption needs to be considered (*i.e.* separation through membranes), due to the relatively high concentrations of adsorbates, interactions between molecules can play a significant role in terms of ‘speeding up’ or ‘slowing down’ other components. In the Maxwell–Stefan approach besides the interaction (or ‘friction’) of the individual molecule with pore walls, also the interaction between the different diffusing molecules is accounted for and balanced with the driving force for mass transport:10
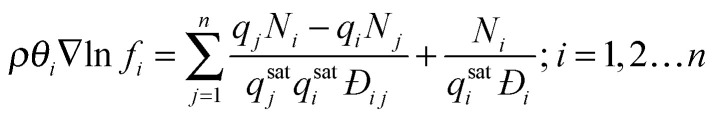
 Within this approach the estimation of Đ_*ij*_ can be difficult, however, a reasonable estimation can be made through a logarithmic (‘Vignes’) interpolation^63,65,66^ based on the single component exchange diffusivities and a correction factor *F* for the confinement of the molecules in the narrow zeolite pores.^67^

For a single component system of tagged and untagged species the saturation capacities are equal and one can show^67^ that the single component exchange coefficient is related to the self diffusivity and MS diffusivity as11
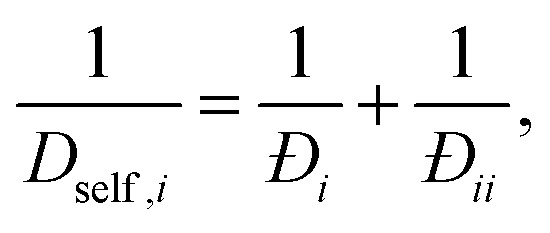
12
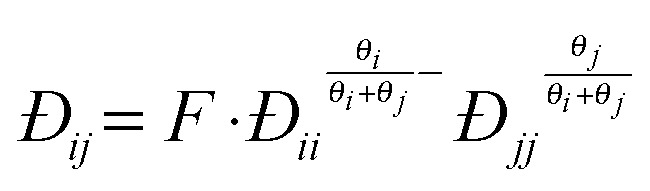
 For mesoporous systems the factor *F* equals 1, while for the microporous materials values <1 hold.^67^

It is evident that in the case of mixture diffusion an accurate estimation of the individual component loading and the driving force is required to satisfactory model such a system. For zeolitic and MOF systems, IAST^68^ provides an acceptable mixture prediction based on the single component isotherms,^62,69,70^ but when adsorption heterogeneity becomes manifest IAST also tends to fail.^71^

At significant loading the molecular interaction can play an important role, strongly influencing the reactant and product concentration profiles. When the loading is relatively low the cross-correlation effects can often be ignored, *i.e.* the system can be modelled as single component system (eqn (8)).

Once diffusion and adsorption for both components have been defined, a model able to describe transport through the composite can be established. For instance, the Maxwell model can be used to describe the effective molar flux (*N*_eff_) of a gas species in a MMM for a suspension of spherical filler particles in a continuous polymer matrix as:^59,72,73^13
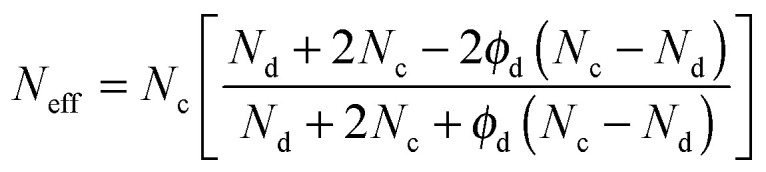
 In this expression, *N*_c_ and *N*_d_ represent the molar fluxes in the continuous and dispersed phases, respectively, and *ϕ*_d_ is the volume fraction of the dispersed phase. The Maxwell model combines flux through the polymer and filler in parallel and series pathways, similar to electrical circuits. The Maxwell model is intended to be applicable for low filler loadings since it assumes that the streamlines associated with diffusive mass transport around filler particles are not affected by the presence of nearby particles. The Bruggeman model,^58,59^ is an improved version of the Maxwell model by accounting for these effects and defines for spherical particles the effective flux in an implicit relation:14
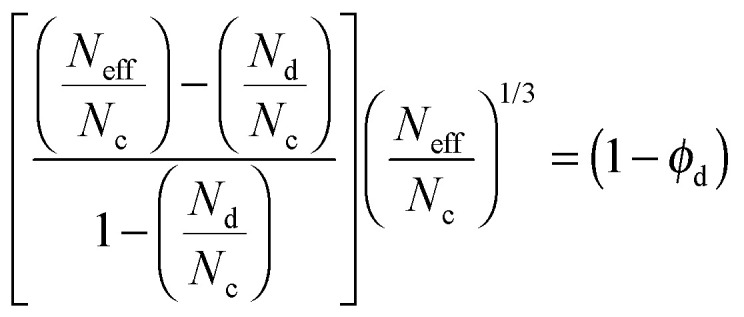
 The Maxwell and Bruggeman models give similar results up to *ϕ*_d_ = 0.2.^58^ Both models describe the permeation of a pure gas through a membrane. Once the effective permeabilities of two gas species are calculated, the ideal selectivity, *S*_*ij*_, the ratio of pure gas permeabilities of each species, can be determined.

Modeling mixture permeation through MMMs is more complicated than describing pure gas permeation since the gas permeabilities of each species can be affected by competition effects between the two species. The most widely applied method for calculating mixture permeation is the so-called dual mode/partial immobilization model.^59,74^ The model proposes that sorption can occur in either the Langmuir or the Henry's Law regime (*i.e.* dual mode sorption) and that the diffusion through these regimes can also be different (partial immobilization). This approach is only based on parameters supplied by pure gas measurements. In this case, the generalized expressions for the permeability coefficients of species *i* and *j* in a binary gas mixture with a vacuum downstream are:15
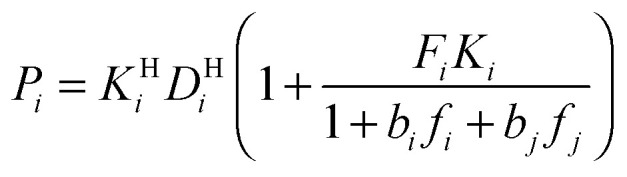
16
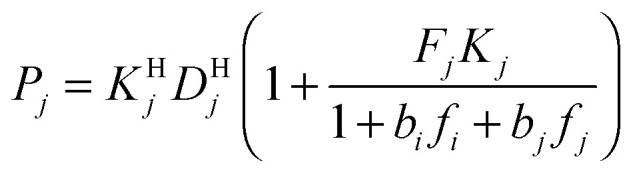
where 
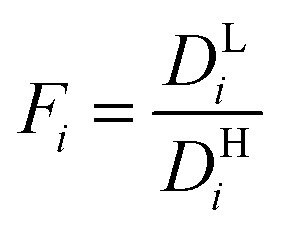
, 
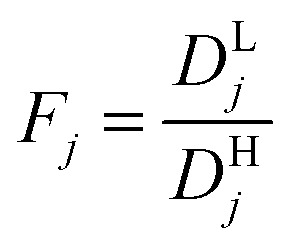
, 
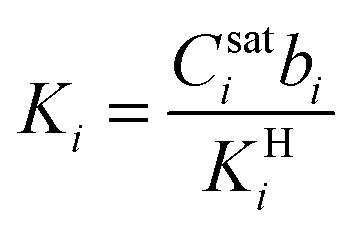
, 
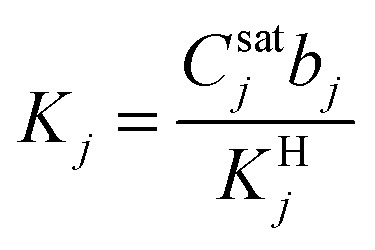
, and *f*_*i*_ and *f*_*j*_ correspond to the upstream fugacities of components *i* and *j*. In these expressions, *D*^H^_*i*_ and *D*^L^_*i*_ are the diffusivities of species *i* in the Henry and Langmuir environments, respectively; *K*^H^_*i*_ is the Henry adsorption coefficient of species *i*, and *C*^sat^_i_and *b*_*i*_ are the Langmuir capacity constant and affinity constant for species *i*, respectively.

When it comes to MOF-MMMs, performance modeling has hardly been explored, mostly a post analysis was performed. Keskin and Sholl studied MMMs consisting of Matrimid® and MOF-5 by using Maxwell and Bruggeman permeation models to predict single gas permeabilities for low and high filler loadings respectively. To calculate mixture permeation, the authors applied a dual mode/partial immobilization method to describe gas transport through MMMs containing IRMOF-1 in Matrimid®. The performance of Cu(hfipbb)(H_2_hfipbb)_0.5_ MMMs was predicted using Maxwell and Bruggeman models.

They illustrated that 20 wt% Cu(hfipbb)(H_2_hfipbb)_0.5_ was enough to bring the MMM above the Robeson's upper bound with a CO_2_/CH_4_ selectivity of 72 and CO_2_ permeability of 15.7 Barrer. Keskin and co-workers have further expanded their modelling activities by combining molecular simulations to predict MOF and polymer properties and Maxwell, extended Maxwell and modified Maxwell models to predict M^4^ performance for a variety of systems comprising ZIFs and most popular MOFs.^72,76–79^

**Fig. 4:**
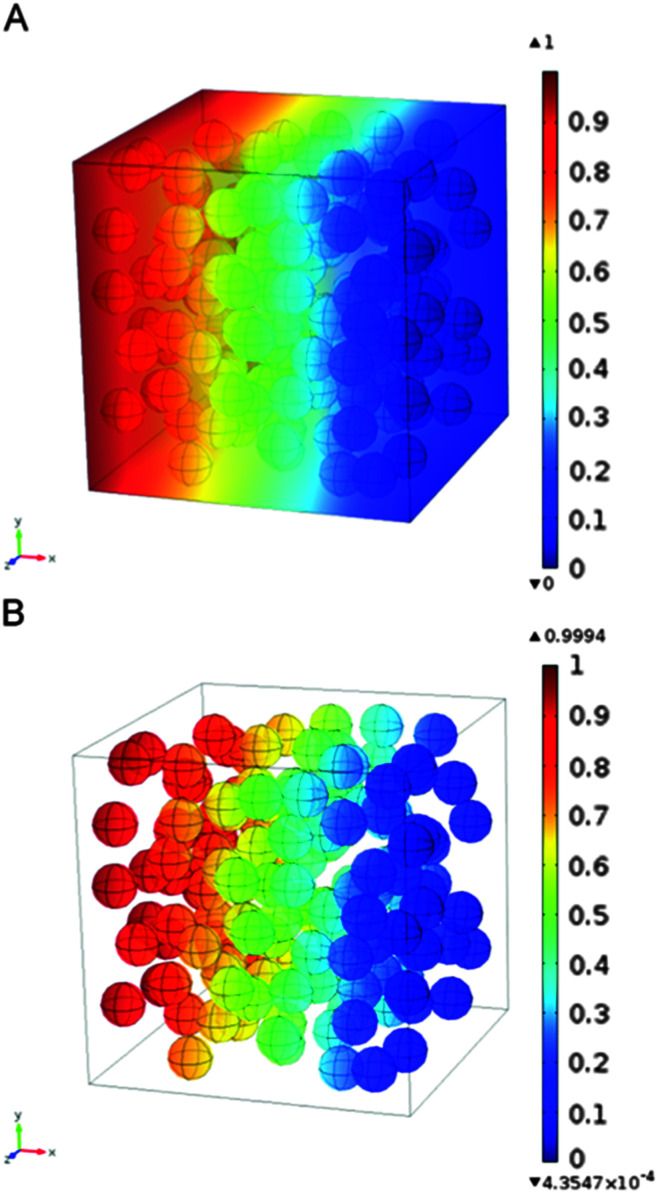
Concentration profiles of (a) both matrix and filler domains, and (b) filler domain only, of a membrane model with randomly distributed filler. Length units are in μm and concentration in mol m^−1^.^75^ Reproduced with permission from Elsevier.

More recently, Nair and co-workers developed a completely new approach for the simulation of MMMs by constructing detailed and large-scale 3D mixed-matrix membrane (MMM) models, which were then solved by finite-element methods (see Fig. 4).^75^ Such models explicitly account for the effects of matrix-filler interfacial equilibrium in addition to the differences in Fickian diffusivity between the two phases. By doing so, they demonstrated that the individual values of the interfacial equilibrium constant or partition coefficient, *K*, the equilibrium ratio between the concentration in the filler and the polymer, and the diffusivity ratio of the filler and the matrix, *D*_f_/*D*_m_, and not the combined permeability ratio *P*_f_/*P*_m_, determine the MMM permeability. This is in contrast to most commonly applied analytical equations (*e.g.*, Maxwell model) that can only predict the MMM permeability under an implicit assumption that *K* and *D*_f_/*D*_m_ can be lumped into a single parameter, the permeability ratio *P*_f_/*P*_m_ = *KD*_f_/*D*_m_. This approach certainly looks like the way to go for modeling of these complex composites.

## C Challenges and targets in developing membranes for post- and pre-combustion CO_2_ capture

The main challenge in post-combustion CO_2_ capture is the low partial pressure of CO_2_ and the huge amount of the flue gas to process. The CO_2_ content (volume basis) can be as low as 4% in a gas turbine plant, around 15% for coal power plants, and more concentrated (∼20–30%) for cement and steel production plants. This low CO_2_ partial pressure represents an enormous challenge for any CO_2_ capture technology: in the case of adsorption and absorption based processes, the use of adsorbents and absorbents with very high affinities is necessary, making regeneration very energy intensive.^80^ In the case of membranes, the driving force (ratio of feed to permeate partial pressure) becomes the limiting parameter, while no regeneration is needed. The routes to increase the driving force of the process are: (i) pressurizing the feed stream, (ii) applying partial vacuum on the permeate side of the membrane or (iii) using a sweep gas on the permeate side of the membrane module.

**Fig. 5:**
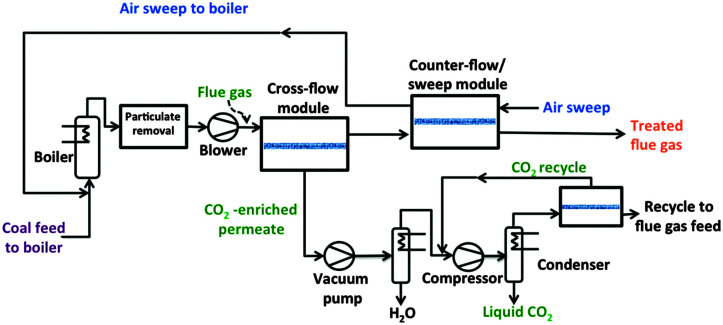
Simplified flow diagram of a two-step counter-flow/sweep membrane process to capture and sequester CO_2_ in flue gas from a coal-fired power plant.^82^

Favre *et al.*^9,81^ have shown that the energy penalty for carbon capture is reduced if membranes with higher selectivities are used, especially when flue gases with high CO_2_ concentrations are involved (*i.e.* biogas combustion). Indeed, several researchers have demonstrated that a single stage membrane process can fulfil the targets for a lower energy penalty if the CO_2_ concentration is higher than 50%. In contrast, when it comes to lower CO_2_ concentrations, multi-stage membrane configurations are needed: Merkel *et al.*^82,83^ compared different multi stage membrane configurations (cross-flow, counter-flow and sweep flow) using a pressure ratio of 5, gas composition data from a 600 MWe coal-fired power plant (11 vol% CO_2_) and the MTR's membrane Polaris® as the base case (Permeance 1000 GPU, CO_2_ selectivity *α* = 50). For the optimal configuration (two-step counter-flow/sweep membrane process, see Fig. 5), a 90% CO_2_ recovery can be achieved at a price of 18 € per ton CO_2_ (including compression). This analysis stresses the importance of advanced engineering analysis in parallel to membrane development: a four-fold reduction in membrane area could be achieved by proper process design.^82^ Similar conclusions were reached by Ramasubramanian and coworkers^84^ using a cheaper, more permeable (3000 GPU) and more selective membrane than Polaris®. In the latter case, it is possible to reach a similar separation target at feed pressures close to 1 bar with multi stage air sweep process, in good agreement with previous results.^85^ A similar approach was followed by Koros *et al.*^86^ employing asymmetric hollow fiber modules instead of the spiral wound modules considered by Merkel and Ramasubramania.^82,84^ Koros and coworkers developed asymmetric hollow fibers from a highly permeable glassy polymer and investigated the performance of modules of these fibers for the same process configuration.^86^ The results show that although hollow fiber modules can be more expensive than spiral wound ones, in terms of CO_2_ capture cost both membrane configurations are comparable: the authors concluded that if hollow fibers (HFbs) can be produced with a permeance higher than 1000 GPU and a moderate selectivity (∼20), their modules can reduce the CO_2_ capture cost to less than 18 € per ton (including compression). As a rough calculation, 200 Barrer would be equivalent to ∼1300 GPU for membranes with a selective layer of 150 nm. This means that membranes with *P*_CO_2__ > 500 Barrer (equivalent to 3300 GPU for 150 nm thick HFbs) and selectivities in the range of 30–40 will certainly achieve the SET objective of 90% CO_2_ capture at a cost below 25 € per MW h: such membranes will result in CO_2_ capture costs below 15 € per ton CO_2_ which, depending on the energy plant, would lower the CO_2_ capture cost to less than 10 € per MW h.

In the case of pre-combustion CO_2_ capture, the application of membranes offers several advantages: (i) the mixture of CO_2_ and H_2_ following the shift reactor is already at high pressure, unlike post-combustion applications and (ii) the application of selective H_2_ permeable membranes can deliver CO_2_ at high pressure, greatly reducing compression costs, while subsequent combustion of H_2_ to produce electricity does not require high pressures. However, in terms of membrane performance (see Fig. 3) separation is very challenging, since achieving H_2_/CO_2_ selectivities higher than 10 implies very low permeances, unsuitable for the treatment of large effluent amounts. For pre-combustion CCS, the gas transport performance of the polymers at elevated temperatures (150–250 °C) is more important than the ambient temperature data used on the Robeson plot. Separation at high temperatures is preferred in order to increase membrane selectivity towards hydrogen. However, only a few systematic gas permeability studies have been performed with polymer containing membranes at higher temperatures and only a couple of studies involved M^4^s (*vide infra*). Currently, the metal supported polybenzimidazole (PBI) membrane under development at DOE's Los Alamos National Laboratory (LANL) represents the state of the art polymeric H_2_ separating membrane for pre-combustion CO_2_ capture.^87,88^ PBI has attracted attention due to its thermal stability and good processability but it has poor performance at 30 °C (*P*_H_2__ = 1 Barrer; *α*(H_2_/CO_2_) = 15), although this improves markedly at 250 °C (*P*_H_2__ = 100 Barrer; *α*(H_2_/CO_2_) = 22). The LANL study shows that more permeable PBI derivatives also display much improved performance at elevated temperatures. For example, the permeability and selectivity of 6F-PBI at 30 °C (*P*_H_2__ = 250 Barrer; *α*(H_2_/CO_2_) = 1.5) is greatly improved at 250 °C (*P*_H_2__ = 1000 Barrer; *α*(H_2_/CO_2_) = 6). In a separate programme of research, MTR's proprietary polymer membrane, Proteus™, displays very promising performance at 150 °C (*P*_H_2__ = 600 Barrer; *α*(H_2_/CO_2_) = 15). Spiral-wound membranes based on this polymer have been the focus of a successful pilot-scale CCS trial that demonstrated good performance over several weeks of operation. Recently Ku *et al.*^89^ published a detailed study on membrane performance requirements for pre-combustion CO_2_ capture applying a single step high temperature membrane process. In electricity generation applications, the permeate stream is combusted to produce power. Gas turbines capable of accepting feed streams with up to 45 vol% hydrogen have been in operation for over 10 years, with more than 80 000 h of operation of the fleet leader. This can be used to advantage in membrane systems, by using the N_2_ as a sweep gas to increase the separation driving force. For membranes displaying higher permeances than 1000 GPU, the estimated H_2_/CO_2_ membrane selectivity requirement for IGCC with 90% CO_2_ capture ranged from about 20 to 60, considerably lower than for industrial H_2_ production. This is not surprising given the ultra-high purity requirement (99.999%) for the latter case. Ideally, membranes displaying such permeances and separation factors larger than 60 would allow high H_2_ recovery rate (>90%). At a 90% overall CO_2_ capture rate, the required H_2_/CO_2_ selectivity drops from about 60 to about 20 as the H_2_ recovery is reduced from 90% to 70%. In summary, high performance membranes will support pre-combustion CCS at an efficiency that matches the targets of 90% carbon capture with only 10% extra cost.^90^ Hence, the development of membranes with target properties of *P*_H_2__ > 500 Barrer and *α*(H_2_/CO_2_) > 30 at >150 °C will allow the SET targets to be reached or exceeded. A permeability of 500 Barrer would be equivalent to a permeance of 1600 GPU for membranes with a selective layer of 300 nm. In case of pre-combustion capture, in view of the envisaged higher temperature and pressure operation, slightly thicker membranes than for post-combustion capture should be used.

## D MOF based mixed matrix membranes for gas separation

**Fig. 6:**
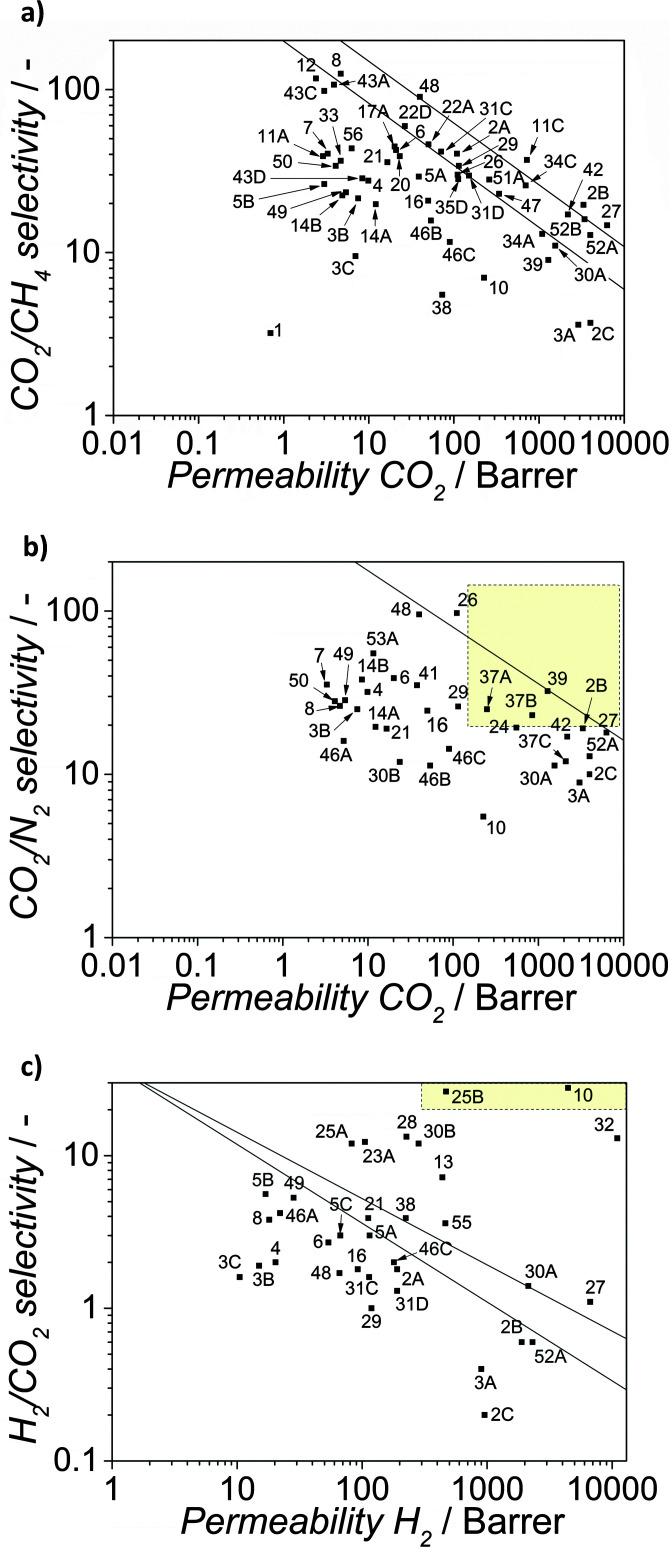
Robeson plots for the separation of CO_2_ from CH_4_ (a) CO_2_ from N_2_ (b) and H_2_ from CO_2_ (c). The graphs contain the most relevant results reported in literature for M^4^s. See Table 2 for references.

As already discussed above, the use of MOFs as fillers offers potential advantages over other porous materials mostly due to: (i) the better affinity of the polymer chains for MOFs in comparison to other inorganic fillers due to their partially organic nature, helping to avoid the so-called “sieve-in-a-cage morphology”,^38,91,92^ the most common MMM deficiency; and (ii) their easily adjustable cavities in terms of size, shape and chemical functionalities that can be tuned by choosing the appropriate ligands in the synthesis^93^ or by post-synthetic functionalization.^94^ Furthermore, when comparing MOFs with other fillers, it needs to be considered that MOFs commonly have a higher pore volume and a lower density than zeolites, meaning that their effect on the membrane properties can be larger for a given weight percentage of the filler. Table 2 summarizes most of the publications on the topic, while in Fig. 6 the reported results have been plotted in the shape of Robeson plots for the most relevant CO_2_ capture related gas pairs.

**Table 2:** Overview of the reported MOF-containing MMMs for gas separation in chronological order

M^4^		wt% loading (best MMM performance)^b^	Example (best performance)^c^						Graph code^d^	Type of analysis	Operation conditions (optimal value)^e^		Published year and ref.
MOF^a^	Polymer^a^		P CO_2_ (Barrer)	CO_2_/CH_4_ selectivity (—)	P CO_2_ (Barrer)	CO_2_/N_2_ selectivity (—)	P H_2_ (Barrer)	H_2_/CO_2_ selectivity (—)			*T* (°C)	Δ*P* (bar)	
Cu 4,4′-BPDC-TED	PAET	10–30 (30)	1.4– (0.7)	18.0– (3.2)	—	—	—	—	[1]	Single gas CO_2_, O_2_, N_2_, CH_4_	25	2	2004^48^
			—	—	—	—	—	—	—	Gas mixture CH_4_/CO_2_ (10 : 90)			

[Cu_2_(PF_6_)(NO_3_)(4,4′-bpy)_4_]2PF_6_·2H_2_O	PSF	2.5–5 (5)	—	—	—	—	—	—	—	Single gas He, H_2_, O_2_, N_2_, CH_4_	35	1	2005^95^

[Zn_2_(1,4-bdc)_2_ (dabco)]·4DMF·0.5H_2_O	PAI	30	46.7– (109)	49.7– (40.4)	46.7– (109)	28.3– (24.6)	79.2– (191)	1.7– (1.8)	[2A]	Single gas	—	—	2006^110^
	6FDA-4MPD		1000– (3330)	23.0– (19.6)	1000– (3330)	21.4– (19.1)	743– (1890)	0.7– (0.6)	[2B]				
	PDMS		2830– (4010)	3.4– (3.7)	2830– (4010)	10.5– (10.0)	673– (955)	0.2– (0.2)	[2C]				

HKUST-1	PDMS	10–40 (30, 10, 40)	2500– (2900)*	3.1– (3.6)*	2500– (3050)*	7.0– (8.9)*	550– (900)*	0.2– (0.4)*	[3A]	Single gas H_2_, CO_2_, O_2_, N_2_, CH_4_	—	—	2006^96^
	PSF	5–10 (5, 10)	6.5– (7.5)*	18.0– (21.5)*	6.5– (7.5)*	20.0– (25.0)*	9.8– (15.0)*	1.5– (1.9)*	[3B]				
Mn(HCOO)_2_	PSF	5–10 (10, 5)	6.5– (7.0)*	18.0– (9.5)*	6.5– (7.0)*	20.0– (25.5)*	9.5– (10.5)*	1.5– (1.6)*	[3C]				

Cu-4,4′-BPY-HFS	Matrimid®	10–40 (20, 30)	7.3– (9.9)	34.7– (27.6)	7.3– (9.9)	33.1– (31.9)	17.5– (20.3)	2.4– (2.0)	[4]	Single gas H_2_, CO_2_, O_2_, N_2_, CH_4_	35	2	2008^101^
		20	—	36.3– (20.5)	—	—	—	2.6– (2.6)	—	Gas mixture H_2_/CO_2_ (50 : 50, 75 : 25) CO_2_/CH_4_ (50 : 50, 10 : 90) CH_4_/N_2_ (94 : 6, 50 : 50)			

IRMOF-1	Matrimid®	20	10.0– (38.8)	28.2– (29.2)	—	—	33.1– (114.9)	3.3– (3.0)	[5A]	Single gas H_2_, CO_2_, CH_4_	50	7	2009^111^
	Ultem®	10, 20 (20)	2.0– (3.0)	30.3– (26.3)	—	—	11.2– (16.9)	5.7– (5.7)	[5B]				
HKUST-1	Matrimid®	30	10.0– (22.1)	28.2– (29.8)	—	—	33.1– (66.9)	3.3– (3.0)	[5C]				

MOF-5	Matrimid®	10–30 (30)	9.0– (20.2)	41.7– (44.7)	9.0– (20.2)	36.0– (38.8)	24.4– (53.8)	2.7– (2.7)	[6]	Single gas H_2_, CO_2_, O_2_, N_2_, CH_4_	35	2	2009^102^
		30	—	38.0– (29.0)	—	—	—	2.3– (2.3)	—	Gas mixture H_2_/CO_2_ (75 : 25, 50 : 50, 25 : 75) CH_4_/N_2_ (94 : 6, 50 : 50, 25 : 75) CO_2_/CH_4_ (10 : 90, 50 : 50, 25 : 75)			

ZIF-8	PPEES	10–30 (30)	6– (25)	—	6– (25)	—	—	—	—	—	30	1, 2, 3, 5, 7, 10	2010^112^

Cu 1,4-BDC	PVAc	15	2.4– (3.3)	34.9– (40.4)	2.4– (3.3)	32.1– (35.4)	—	—	[7]	Single gas He, CO_2_, O_2_, N_2_, CH_4_	35	4.5 (0.1 for CO_2_)	2010^100^

ZIF-8	Matrimid®	20–60 (50)	9.5– (4.7)	39.7– (124.9)	9.5– (4.7)	30.6– (26.2)	28.9– (18.1)	3.0– (3.8)	[8]	Single gas H_2_, CO_2_, O_2_, N_2_, CH_4_, C_3_H_8_	25	2.7	2010^103^
		50–60 (50, 60)	—	42.1– (89.2)	—	—	—	2.6– (7.0)	—	Gas mixture H_2_/CO_2_ (50 : 50) CO_2_/CH_4_ (10 : 90)			

HKUST-1	Matrimid®	10–30 (30)	10.0– (17.5)* (GPU)	18.0– (24.0)*	11.0– (18.5)* (GPU)	23.5– (24.5)*	—	—	[9]	Gas mixture CO_2_/CH_4_ (10 : 90, 35 : 65, 75 : 25) CO_2_/N_2_ (10 : 90, 35 : 65, 75 : 25)	35	10	2010^113^

HKUST-1	PMDA-ODA	3–6 (3, 6)	306.6– (227.2)	12.0– (7.0)*	306.6– (227.2)	8.0– (5.5)*	3066– (4445)	10.0– (27.8)	[10]	Single gas H_2_, CO_2_, O_2_, N_2_, CH_4_	25	10	2010^114^

ZIF-90	Ultem®	15	1.4– (2.9)*	38– (39)*	—	—	—	—	[11A]	Single gas CO_2_, CH_4_	35	4.5	2010^40^
	Matrimid®		7.5– (10.5)*	34– (35)*	—	—	—	—	[11B]				
	6FDA-DAM		390– (720)	24– (37)	—	—	—	—	[11C]	Gas mixture CO_2_/CH_4_ (50 : 50)	25	2	

ZIF-20	PSF	8	—	—	—	—	—	—	—	Gas mixture O_2_/N_2_ (50 : 50)	35	2	2011^98^

NH_2_-MIL-53(Al)	PSF	8, 16, 25, 40 (25)	2.0– (2.4)	45– (117)	—	—	—	—	[12]	Gas mixture CO_2_/CH_4_ (50 : 50)	−10, 35	1, 3, 5, 7, 10, 13	2011^45^

ZIF-7	PBI	10, 25, 50 (50)	—	—	—	—	3.7– (26.2)	8.7– (14.9)	—	Single gas H_2_, CO_2_	35	3.5	2011^115^
							75– (440)*	8.5– (7.2)*	[13]	Gas mixture H_2_/CO_2_ (50 : 50)	35, 60, 80, 120, 150, 180	7	

ZIF-8 + S1C	PSF	16 + 0, 8 + 8 (16 + 0)	4.6– (12.1)	24.3– (19.8)	5.9– (12.3)	24.6– (19.5)	—	—	[14A]	Gas mixture CO_2_/CH_4_ (50 : 50) CO_2_/N_2_ (50 : 50) O_2_/N_2_ (50 : 50) H_2_/CH_4_ (50 : 50)	35	2	2011^97^
HKUST-1 + S1C		16 + 0, 8 + 8 (8 + 8)	4.6– (4.9)	24.3– (22.4)	5.9– (8.4)	24.6– (38.0)	—	—	[14B]				
HKUST-1	Matrimid®	10, 20, 30 (30)	10.0– (17.5)* (GPU)	18.5– (23.0)*	11.5– (19.5)* (GPU)	18.0– (23.5)*	—	—	[15A]	Gas mixture CO_2_/CH_4_ (10 : 90, 35 : 65, 75 : 25) CO_2_/N_2_ (10 : 90, 35 : 65, 75 : 25)	35	10	2011^116^
ZIF-8			10.0– (22.5)* (GPU)	18.5– (19.5)*	11.5– (20.0)* (GPU)	18.0– (19.5)*	—	—	[15B]				
MIL-53(Al)			10.0– (20.0)* (GPU)	18.5– (22.5)*	11.5– (20.0)* (GPU)	18.0– (23.0)*	—	—	[15C]				

ZIF-8	PPEEs	10, 20, 30 (30)	5.4– (50.0)	22.9– (20.8)	5.4– (50.0)	30.1– (24.5)	6.7– (92.3)	1.3– (1.8)	[16]	Single gas H_2_, CO_2_, O_2_, N_2_, CH_4_, C_2_H_4_, C_2_H_6_	10, 20, 30, 40	1	2011^99^

ZIF-8	6FDA-DAM	16.4, 28.7, 48 (48)	—	—	—	—	—	—	—	Single gas C_3_H_6_, C_3_H_8_	35	2	2012^117^
										Gas mixture C_3_H_6_/C_3_H_8_ (50 : 50)		1.4, 2.8, 4.1, 5.5	

MIL-101(Cr)	PSF	8, 16, 24	—	—	—	—	—	—	—	Single gas O_2_, N_2_	30	3	2012^118^
MOF-508a(Zn)		8	—	—	—	—	—	—	—				
MIL-53(Al)			—	—	—	—	—	—	—				
MIL-100(Fe)			—	—	—	—	—	—	—				

MIL-53(Al)	6FDA-ODA	25	14.5– (21.0)*	48.0– (44.0)*	—	—	—	—	—	Single gas CO_2_, CH_4_	35	10	2012^122^
			14.5– (21.0)*	42.0– (42.5)*	—	—	—	—	[17A]	Gas mixture CO_2_/CH_4_ (50 : 50)			
NH_2_-MIL-53(Al)		10, 15, 20, 25, 30, 32, 35 (32)	14.5– (14.7)*	48.0– (76.0)*	—	—	—	—	—	Single gas CO_2_, CH_4_			
			14.5– (14.7)*	42.0– (53.0)*	—	—	—	—	[17B]	Gas mixture CO_2_/CH_4_ (50 : 50)			

ZIF-8	Ultem®	10, 13 (13)	—	—	14.0– (26.0) (GPU)	30.0– (36.0)	—	—	—	Single gas CO_2_, N_2_	25, 30, 35, 45	6.7	2012^119^
			—	—	(26.0)* (GPU)	(32.0)	—	—	[18]	Gas mixture CO_2_/N_2_ (20 : 80)	25, 35, 45	1.4, 2.1, 2.8, 3.4	

MIL-53(Al)	PMDA-ODA	5	0.30– (0.21) (GPU)	72.1– (50.5)	0.30– (0.21) (GPU)	34.8– (27.5)	0.35– (0.42) (GPU)	1.1– (2.0)	[19A]	Single gas He, H_2_, CO_2_, N_2_, CH_4_	25	6	2012^120^
MOF-5			0.30– (0.27) (GPU)	72.1– (56.8)	0.30– (0.27) (GPU)	34.8– (14.1)	0.35– (0.24) (GPU)	1.1– (0.9)	[19B]				
HKUST-1			0.30– (0.32) (GPU)	72.1– (73.6)	0.30– (0.32) (GPU)	34.8– (38.1)	0.35– (0.44) (GPU)	1.1– (1.3)	[19C]				

ZIF-8	Matrimid®	10, 25 (25)	10.7– (23.2)	34– (39)	—	—	—	—	[20]	Single gas CO_2_, CH_4_	35	4.5	2012^121^

ZIF-8	Matrimid®	5, 10, 20, 30, 40 (20, 30)	8.1– (16.6)	35.2– (35.8)	8.1– (16.6)	22.4– (19.0)	32.7– (112.1)	4.0– (3.9)	[21]	Single gas H_2_, CO_2_, O_2_, N_2_, CH_4_	22	4	2012^122^

UiO-66	6FDA-ODA	25	14.4– (50.4)	44.1– (46.1)	—	—	—	—	[22A]	Single gas CO_2_, CH_4_	35	10	2012^123^
			—	41.7– (42.3)	—	—	—	—	—	Gas mixture CO_2_/CH_4_ (50 : 50)			
NH_2_-UiO-66			14.4– (13.7)	44.1– (51.6)	—	—	—	—	[22B]	Single gas CO_2_, CH_4_			
			—	41.7– (44.7)	—	—	—	—	—	Gas mixture CO_2_/CH_4_ (50 : 50)			
HKUST-1			14.4– (21.8)	44.1– (51.2)	—	—	—	—	[22C]	Single gas CO_2_, CH_4_			
			—	41.7– (50.7)	—	—	—	—	—	Gas mixture CO_2_/CH_4_ (50 : 50)			
NH_2_-HKUST-1			14.4– (26.6)	44.1– (59.6)	—	—	—	—	[22D]	Single gas CO_2_, CH_4_			
			—	41.7– (52.4)	—	—	—	—	—	Gas mixture CO_2_/CH_4_ (50 : 50)			
UiO-67			14.4– (20.8)	44.1– (15.0)	—	—	—	—	[22E]	Single gas CO_2_, CH_4_			
			—	41.7– (15.0)	—	—	—	—	—	Gas mixture CO_2_/CH_4_ (50 : 50)			

ZIF-8	PBI	18, 20, 29, 34, 59 (29)	—	—	—	—	3.7– (105.4)	8.6– (12.3)	[23A]	Single gas H_2_, CO_2_	25	3.5	2012^124^
	PBI/Matrimid®	10, 20, 33 (10)	—	—	—	—	2.1– (8.9) (GPU)	6.2– (9.5) (GPU)	—				
		10, 20, 33 (10)	—	—	—	—	(65.4) (GPU)	(12.3) (GPU)	[23B]	Gas mixture H_2_/CO_2_ (50 : 50)	25, 35, 50, 80, 120, 150, 180	7	

ZIF-8	6FDA-DAM : DABA 4 : 1	20	—	—	211.4– (553)	21.3– (19.3)	—	—	[24]	Single gas CO_2_, N_2_	30	1.4	2012^86^

ZIF-8	PBI	30, 60 (30)	—	—	—	—	4.1– (82.5)	8.9– (12.0)	[25A]	Single gas H_2_, CO_2_	35	3.5	2013^107^
			—	—	—	—	(470)	(26.3)	[25B]	Gas mixture H_2_/CO_2_ (50 : 50) H_2_/CO_2_/CO (49.5 : 49.5 : 1)	35, 60, 120, 180, 230	2	

ZIF-7	Pebax®	8, 22, 34 (22)	72– (111)	14– (30)	72– (111)	34– (97)	—	—	[26]	Single gas CO_2_, N_2_, CH_4_	20	6.5 (2.75 for CO_2_)	2013^125^

ZIF-8	PIM-1	13.8, 24.2, 32.4, 39.0 (39.0)	4390– (6300)	14.2– (14.7)	4390– (6300)	24.4– (18.0)	1630– (6680)	0.4– (1.1)	[27]	Single gas He, H_2_, CO_2_, O_2_, N_2_, CH_4_	20–22	1	2013^126^

HKUST-1	P84	20	—	—	—	—	—	—	—	Gas mixture C_2_H_4_/C_2_H_6_ (80 : 20)	—	5, 10, 15	2013^127^
FeBTC			—	—	—	—	—	—	—				
MIL-53(Al)			—	—	—	—	—	—	—				

HKUST-1	P84	10, 20, 40 (20)	—	—	—	—	—	—	—	Gas mixture C_2_H_4_/C_2_H_6_ (80 : 20)	—	5, 10, 15	2013^127^

ZIF-90	PBI	10, 25, 45 (45)	—	—	—	—	4.1– (24.5)	8.9– (25)	—	Single gas H_2_, CO_2_	35	3.5	2013^128^
		45	—	—	—	—	(226.9)	(13.3)	[28]	Gas mixture H_2_/CO_2_ (50 : 50)	35, 60, 80, 120, 180	7	

HKUST-1	PPO	10, 20, 30, 40, 50 (40)	68.7– (115)*	16.4– (34)*	68.7– (115)*	16.0– (26)*	75.0– (119)*	1.1– (1.0)*	[29]	Single gas H_2_, CO_2_, N_2_, CH_4_	30	—	2013^129^

ZIF-8	6FDA-durene	33.3	468.5– (1552.9)	15.6– (11.0)	468.5– (1552.9)	13.4– (11.3)	518.5– (2136.6)	1.1– (1.4)	[30A]	Single gas H_2_, CO_2_, O_2_, N_2_, CH_4_	35	3.5	2013^130^
	6FDA-durene (cross-linked)		0.4– (23.7)	(16.9)	0.4– (23.7)	(11.9)	52.1– (283.5)	130.3– (12.0)	[30B]				

NH_2_-MIL-53(Al)	6FDA : DSDA-4MPD : 4,4′-SDA 1 : 1	0, 5, 10, 15 (15)	57.9– (66.5)	35.1– (36.9)	—	—	90.1– (100)	1.6– (1.8)	[31A]	Single gas H_2_, CO_2_, CH_4_	35	3	2013^131^
	6FDA-4MPD : 4,4′-SDA 1 : 1	10	134– (137)	30.2– (27.2)	—	—	169– (175)	1.3– (1.3)	[31B]				
NH_2_-MIL-101(Al)	6FDA : DSDA-4MPD : 4,4′-SDA 1 : 1	0, 5, 10 (10)	57.9– (70.9)	35.1– (41.6)	—	—	90.1– (114)	1.6– (1.6)	[31C]				
	6FDA-4MPD : 4,4′-SDA 1 : 1	10	134– (151)	30.2– (29.6)	—	—	169– (191)	1.3– (1.3)	[31D]				

NH_2_-CAU-1	PMMA	5, 10, 15, 20, 25 (15)	—	—	—	—	5000– (11 000)	3– (13)	[32]	Single gas H_2_, CO_2_	RT	3	2013^132^
			—	—	—	—	—	2– (10)*	—	Gas mixture H_2_, CO_2_ (—)			

MIL-68(Al)	PSF	4, 8 (8)	5.4– (4.7)	31.1– (36.5)	—	—	—	—	[33]	Gas mixture CO_2_/CH_4_ (50 : 50)	35	2	2013^131^

HKUST-1	PLLA	5	—	—	—	—	—	—	—	–CO_2_, O_2_	23	—	2013^133^

ZIF-8	6FDA-durene (400 °C)	20	541– (1090)	13.1– (13.0)	—	—	—	—	[34A]	Single gas CO_2_, CH_4_, C_3_H_6_, C_3_H_8_	35	10 (3.5 for C_3_H_6_ and C_3_H_8_)	2013^134^
	6FDA-durene : DABA 9 : 1 (200 °C)	5, 10, 15, 20, 30, 40 (40)	256– (779)	19.5– (20.9)	—	—	—	—	[34B]				
	6FDA-durene : DABA 7 : 3 (400 °C)	20	429– (698)	26.0– (25.8)	—	—	—	—	[34C]				
	6FDA-durene : DABA 9 : 1 (400 °C)	20, 40 (20)	305– (728)	13.8– (19.6)	—	—	—	—	[34D]	Gas mixture CO_2_/CH_4_ (50 : 50)	35	20	

NH_2_-MIL-53(Al)	Matrimid®	15	6.2– (9.2)	31.0– (2.1)	—	—	—	—	[35A]	Single gas CO_2_, CH_4_	35	10	2013^135^
			—	28.5– (2.1)	—	—	—	—	—	Gas mixture CO_2_/CH_4_ (50 : 50)			
	Ultem®	15	1.5– (3.0)	39.5– (36.2)	—	—	—	—	[35B]	Single gas CO_2_, CH_4_			
			—	31.6– (36.1)	—	—	—	—	—	Gas mixture CO_2_/CH_4_ (50 : 50)			
	6FDA-ODA : DAM 1 : 1	15, 20, 22 (10)	54.1– (51.2)	23.5– (34.1)	—	—	—	—	[35C]	Single gas CO_2_, CH_4_			
			—	23.6– (31.8)	—	—	—	—	—	Gas mixture CO_2_/CH_4_ (50 : 50)			
	6FDA-ODA : DAM 1 : 4	10, 15, 20 (15)	130.0– (113)	23.2– (28.2)	—	—	—	—	[35D]	Single gas CO_2_, CH_4_			
			—	23.6– (28.5)	—	—	—	—	—	Gas mixture CO_2_/CH_4_ (50 : 50)			
	6FDA-ODA : DAM 1 : 1 (APTMDS)	15, 20, 25, 30, 32, 35 (30)	32.2– (58.5)	18.9– (36.6)	—	—	—	—	[35E]	Single gas CO_2_, CH_4_			
			—	20.2– (33.9)	—	—	—	—	—	Gas mixture CO_2_/CH_4_ (10 : 90) CO_2_/CH_4_ (35:65) CO_2_/CH_4_ (50 : 50) CO_2_/CH_4_ (60 : 40) CO_2_/CH_4_ (80 : 20) CO_2_/CH_4_ (85 : 15)	30, 35, 45, 60, 75	10	

MIL-53	Matrimid®	15	6.2– (6.7)	31.0– (9.4)	—	—	—	—	[35F]	Single gas CO_2_, CH_4_	35	10	2013^135^
			—	28.5– (8.5)	—	—	—	—	—	Gas mixture CO_2_/CH_4_ (50 : 50)			
	Ultem®	15	1.5– (1.8)	39.5– (43.1)	—	—	—	—	[35G]	Single gas CO_2_, CH_4_			
			—	31.6– (42.8)	—	—	—	—	—	Gas mixture CO_2_/CH_4_ (50 : 50)			
	6FDA-ODA : DAM 1 : 1	20	54.1– (61.5)	23.5– (12.5)	—	—	—	—	[35H]	Single gas CO_2_, CH_4_			
			—	23.6– (13.0)	—	—	—	—	—	Gas mixture CO_2_/CH_4_ (50 : 50)			
	6FDA-ODA : DAM 1 : 4	25	130.0– (123)	23.2– (18.1)	—	—	—	—	[35I]	Single gas CO_2_, CH_4_			
			—	23.6– (19.1)					—	Gas mixture CO_2_/CH_4_ (50 : 50)			
	6FDA-ODA : DAM 1 : 1 (APTMDS)	25	32.2– (76.4)	18.9– (8.9)					[35J]	Single gas CO_2_, CH_4_			
			—	20.2– (8.8)	—	—	—	—	—	Gas mixture CO_2_/CH_4_ (50 : 50)			

TKL-107	Matrimid®	5, 10, 20, 30 (20)	7– (17)*	36– (64.6)	—	—	—	—	—	Single gas CO_2_, CH_4_	25	2	2013^136^
			6– (15)*	24– (50.3)	—	—	—	—	[36]	Gas mixture CO_2_/CH_4_ (20 : 80)			

CPO-27(Mg)	XLPEO	10	—	—	380– (250)	22– (25)	—	—	[37A]	Single gas CO_2_, N_2_	25	2	2013^137^
	6FDA-TMPDA	10	—	—	650– (850)	14– (23)	—	—	[37B]				
	PDMS	20	—	—	3100– (2100)	9.5– (12)	—	—	[37C]				
													
Silica-(ZIF-8) core–shell	PSF	8, 12, 16, 20, 32 (32)	11.8– (73.1)*	10.2– (5.5)*	—	—	35.0– (224.1)*	3.4– (3.9)*	[38]	Gas mixture H_2_/CO_2_ (50 : 50) CO_2_/CH_4_ (10 : 90, 50 : 50, 90 : 10)	35, 60, 90,120, 150	2	2014^138^
											35, 60, 90, 120		

ZIF-8	Pebax®	5, 10, 15, 20, 25, 30, 35 (35)	351– (1287)	8.3– (9.0)	351– (1287)	33.8– (32.3)	—	—	[39]	Single gas CO_2_, O_2_, N_2_, CH_4_	RT	2, 6	2014^139^
		5, 10, 15, 20, 25, 30, 35, 40, 50 (25)	—	—	200– (900)*	66– (53)*	—	—	—	Gas mixture CO_2_/N_2_ (10 : 90)	25	1.5	

MIL-53	Matrimid®	5, 10, 15, 20 (15)	6.4– (12.4)	28.2– (51.8)	—	—	—	—	[40]	Single gas CO_2_, CH_4_	35	3	2014^140^

ZIF-8	PSF	8	—	—	—	—	—	—	—	Gas mixture H_2_/CH_4_ (50 : 50) O_2_/N_2_ (50 : 50)	35	2	2014^141^
NH_2_-MIL-53(Al)			—	—	—	—	—	—	—				

[Cd_2_6FDA(H_2_O)]_2_5H_2_O	6FDA-ODA	10	20.6– (37.8)	33.1– (44.8)	20.6– (37.8)	26.4– (35.1)	—	—	[41]	Single gas CO_2_, N_2_, CH_4_	25	2	2014^142^

MIL-53(Al)	PMP	5, 10, 15, 20, 25, 30, 35, 40 (30)	—	—	—	—	100– (365)*	0.11– (0.04)	—	Single gas H_2_, CO_2_	30	2, 4, 6, 8	2014^143^

ZIF-8	6FDA-durene	3, 5, 7, 10, 15, 20, 30 (30)	1468.3– (2185.5)	22.6– (17.1)	1468.3– (2185.5)	25.4– (17.0)	—	—	[42]	Single gas CO_2_, O_2_, N_2_, CH_4_	RT	2, 6	2014^144^
			—	—	—	—	—	—	—	Gas mixture CO_2_/N_2_ (10 : 90)	25	1.5	

NH_2_-MIL-53(Al) + MSSs	PSF	16 + 0, 12 + 4, 8 + 8, 4 + 12 (4 + 12)	—	—	—	—	—	—	—	Gas mixture O_2_/N_2_ (50 : 50) H_2_/CH_4_ (50 : 50)	35	2	2014^145^
NH_2_-MIL-53(Al) + MSSs	Matrimid®	8 + 8, 4 + 12 (4 + 12)	—	—	—	—	—	—	—				

NH_2_-MIL-53(Al)	Matrimid®	15, 20, 25 (25)	4.8– (3.9)	100– (107)	—	—	—	—	[43A]	Gas mixture CO_2_/CH_4_ (50 : 50)	0, 25, 35	3, 5, 9, 12	2014^47^
	PSF	15, 20, 25 (25)	5.2– (5.4)	23.0– (27.5)	—	—	—	—	[43B]		35	3	
NH_2_-MIL-101(Al)	Matrimid®	8, 15, 25 (25)	4.8– (3.0)	100– (98)	—	—	—	—	[43C]		0, 25, 35	3, 5, 9, 12	
	PSF	8, 15, 25 (25)	5.2– (8.4)	23.0– (28.5)	—	—	—	—	[43D]		35	3	

ZIF-8	6FDA-DAM	17, 30 (17)	—	—	—	—	—	—	—	Single gas O_2_, N_2_	35	2	2014^146^
										Gas mixture C_3_H_6_, C_3_H_8_ (50 : 50)		1.4	

ZIF-8	Matrimid®	15	9– (26)*	34.5– (35)*	—	—	—	—	—	Single gas CO_2_, CH_4_	35	3.45	2014^147^
ZIF-7-8-(20)			9– (20)*	34.5– (35.5)*	—	—	—	—	—				
ZIF-8-ambz-(15)			9– (12)*	34.5– (36)*	—	—	—	—	—				
ZIF-8-ambz-(30)			9– (11)*	34.5– (38.5)*	—	—	—	—	—				
ZIF-7-8-(20)			8– (19)*	43– (41)*	—	—	—	—	[44A]	Gas mixture CO_2_/CH_4_ (50 : 50)	—	6.9, 13.8, 27.6, 41.4	
ZIF-8-ambz-(15)			8– (14)*	43– (40)*	—	—	—	—	[44B]				
ZIF-8-ambz-(30)			8– (11)*	43– (42.5)*	—	—	—	—	[44C]				

FeBTC	Matrimid®	10, 20, 30	14– (14)*	55– (35)*	—	—	—	—	—	Single gas CO_2_, CH_4_	35	2– 40 (40)	2014^148^
			14– (8.2)*	22– (28)*	—	—	—	—	[45]	Gas mixture CO_2_/CH_4_ (50 : 50)	—	5	

ZIF-8	PBI-BuI	10, 20, 30 (30)	2.3– (5.2)	57.0– (43.6)	2.3– (5.2)	26.8– (16.0)	6.2– (22.1)	2.7– (4.2)	[46A]	Single gas He, H_2_, CO_2_, N_2_, CH_4_	35	20	2014^149^
	DMPBI-BuI	10, 20, 30 (30)	3.8– (53.9)	47.2– (15.7)	3.8– (53.9)	21.7– (11.3)	12.8– (127.5)	3.4– (2.4)	[46B]				
	DBzPBI-BuI	10, 20 (20)	25.8– (89.8)	15.9– (11.6)	25.8– (89.8)	12.9– (14.3)	61.4– (180.3)	2.4– (2.0)	[46C]				

NH_2_-MIL-53(Al)	PMP	5, 10, 15, 20, 25, 30, 35, 40 (30)	96.5– (358.2)	8.8– (24.4)	—	—	—	—	—	Single gas CO_2_, CH_4_	30	2, 4, 6, 8	2014^150^
			80.1– (339.5)	8.1– (22.9)	—	—	—	—	[47]	Gas mixture CO_2_/CH_4_ (10 : 90)			

MIL-53(Al)-ht	Matrimid®	33.3, 37.5 (37.5)	8.4– (51)	39.4– (47.0)	8.4– (51)	33.6– (28.3)	25.7– (103)	3.1– (2.0)	—	Single gas H_2_, CO_2_, O_2_, N_2_, CH_4_	35	2	2014^151^
MIL-53(Al)-as		37.5	8.4– (40)	39.4– (90.1)	8.4– (40)	33.6– (95.2)	25.7– (66.0)	3.1– (1.7)	[48]				

c-MOF-5	PEI	5, 15, 25 (25)	1.7– (5.4)	18.7– (23.4)	1.7– (5.4)	16.8– (28.4)	10.1– (28.3)	6.0– (5.3)	[49]	Single gas H_2_, CO_2_, N_2_, CH_4_	25	6	2014^152^

HKUST-1	Ultem®	10, 20, 30, 35, 40 (35)	1.1– (4.1)	36.8– (34.0)	1.1– (4.1)*	28.0– (28.0)*	—	—	[50]	Single gas CO_2_, O_2_, N_2_, CH_4_	35	3.5	2014^153^

HKUST-1	ODPA-DAM (annealed 200 °C 24 h)	10, 15, 20, 30, 40, 50 (40)	47.7– (260.7)	29– (28)*	—	—	—	—	[51A]	Single gas CO_2_, O_2_, N_2_, CH_4_	35	2	2014^154^
	Matrimid® (annealed 200 °C 24 h)	20	7.6– (24.8)	37.5– (37.8)	—	—	—	—	[51B]				
ZIF-8	ODPA-DAM (annealed 200 °C 24 h)		47.7– (134)*	29– (26)*	—	—	—	—	[51C]				

ZIF-71	6FDA-durene	10, 20, 30 (20)	959– (4006)	16.4– (12.8)	959– (4006)	14.7– (12.9)	756– (2310)	0.8– (0.6)	[52A]	Single gas H_2_, CO_2_, O_2_, N_2_, CH_4_, C_2_H_4_, C_2_H_6_, C_3_H_6_, C_3_H_8_	35	3.5 (2 for C_2_H_4_, C_2_H_6_, C_3_H_6_ and C_3_H_8_)	2014^155^
		10, 20, 30 (20)	917– (3435)	21.8– (16.0)	—	—	—	—	[52B]	Gas mixture CO_2_/CH_4_ (50 : 50)	35	7	

[Cu_2_(Glu)_2_(μ-bpa)]·(CH_3_CN)	POZ	5, 10, 15, 20 (15)	—	—	28– (11.6)*	1– (55)*	—	—	[53A]	Single gas CO_2_, N_2_	—	3.1	2014^156^
[Cu_2_(Glu)_2_(μ-bpp)]·(C_3_H_6_O)			—	—	28– (16.0)*	1– (7)*	—	—	[53B]			0.4	

MIL-53(Al)	Matrimid®	10, 20, 30 (30)	14– (24)*	55– (66)*	—	—	—	—	—	Single gas CO_2_, CH_4_	35	2.5, 5, 7.5, 10, 12.5, 15, 20, 25, 30, 40	2014^157^
ZIF-8			14– (24)*	55– (72)*	—	—	—	—	—				
HKUST-1			14– (18)*	55– (52)*	—	—	—	—	—				
MIL-53(Al)			9– (18)*	5– (40)*	—	—	—	—	[54A]	Gas mixture CO_2_/CH_4_ (50 : 50)		2.5, 5, 7.5, 10, 15, 20	
ZIF-8			9– (20)*	5– (37)*	—	—	—	—	[54B]				
HKUST-1			9– (14)*	5– (46)*	—	—	—	—	[54C]				

ZIF-11	PSF	4.7	—	—	—	—	—	—	—	—	—	—	2014^158^
	PES	4.7	—	—	—	—	—	—	—				
	PBI	16.1, 29.7, 39.5 (39.5)	—	—	—	—	17.2– (464.7)	5.0– (3.6)	[55]	Single gas H_2_, CO_2_	RT	—	

b-Cu 1,4-BDC	Matrimid®	8	5.8– (5.2)	59.8– (45)	—	—	—	—	—	Gas mixture CO_2_/CH_4_ (50 : 50)	25	3, 4.5, 6, 7.5	2014^159^
nc-Cu 1,4-BDC		8	5.8– (5.0)	59.8– (49.4)	—	—	—	—	—				
ns-Cu 1,4-BDC		2, 4, 8 (8)	5.9– (2.8)	47.7– (88.2)	—	—	—	—	—			3, 4.5, 6, 7.5	
ns-Cu 2,6-NDC		8	5.8– (6.3)	59.8– (43.5)	—	—	—	—	[56]			3	

ZIF-8 100 nm	PSF	5	25.7– (15.6) (GPU)	19.4– (28.5)	—	—	—	—	—	Single gas CO_2_, CH_4_	27	4	2014^160^
ZIF-8 300 nm			25.7– (25.9) (GPU)	19.4– (5.8)	—	—	—	—	—				
ZIF-8 500 nm			25.7– (28.1) (GPU)	19.4– (5.8)	—	—	—	—	—				

ZIF-8	PDMS	2.5, 5, 10, 15, 20	—	—	—	—	—	—	—	Single gas C_3_H_8_, N_2_	—	1.5	2015^161^
			—	—	—	—	—	—	—	Gas mixture C_3_H_8_/N_2_ (10 : 90, 20 : 80, 30 : 70, 40 : 60)	20, 27, 35, 45, 55	0.5, 1.5, 2.5, 3.5, 4.5	

a Abbreviations: μ-BPA: 1,2-bis(4-pyridyl) ethane; μ-BPP: 1,3-bis(4-pyridyl)propane; 1,4-BDC: 1,4-benzenedicarboxylate; 2,6-NDC: 2,6-napthalenedicarboxylate; 2-amBzIM: 2-aminobenzimidazole; 4,4′-BPDC: 4,4′-biphenyl dicarboxylate; 4,4′-BPY: 4,4′-bipyridine; 4,4′-SDA: bis(4-aminophenyl) sulphide; 4MPD (or durene): 2,3,5,6-tetramethyl-1,4-phenylenediamine; 6FDA: 4,4′-(hexafluoroisopropylidene)diphthalic anhydride; APTMDS: bis(3-aminopropyl)tetramethyldisiloxane; b: bulk; BuI: 5-*tert*-butylisophthalic acid; BzIM: benzimidazole; DABA: 3,5-diaminobenzoic acid; DABCO: 1,4-diazabicyclo[2.2.2]octane; DAM (or TMPDA): 2,4,6-trimethyl-*m*-phenylenediamine; DBzPBI: PBI after N-substitution reaction with 4-*tert*-butylbenzyl bromide; DMPBI: PBI after N-substitution reaction with methyl iodide; DSDA: 3,3′,4,4′-diphenylsulfone tetracarboxylic dianydride; Glu: glutarate; HFS: hexafluorosilicate; MSS: mesoporous silica spheres; nc: nanoparticle crystals; ns: nanosheets; ODA: 4,4′-oxydianiline; ODPA: 4,4′-oxydiphthalicanhydride; PAET: poly(3-acetoxyethylthiophene); PAI: polyamide-imide; PBI: polybenzimidazole; PDMS: polydimethylsiloxane; PEI: polyetherimide; PES: polyestersulfone; PLLA: poly(l-lactic acid); PMDA: pyromellitic dianhydride; PMMA: poly(methyl methacrylate); PMP: poly(4-methyl-1-pentyne); POZ: polyoxazoline; PPEES: poly(1,4-phenylene ether-ether-sulfone); PPO: poly(2,6-dimethyl-1,4-phenylene oxide); PSF: polysulfone; PVAc: poly(vinyl acetate); TED: triethylenediamine; XLPEO: cross-linked polyethylene oxide.

b Maximum MOF loading in terms of MMM permselectivity performance. Values are given in brackets.

c CO_2_ and H_2_ permeabilities and CO_2_/CH_4_, CO_2_/N_2_ and H_2_/CO_2_ selectivities of the pure polymer and the MMMs with the optimal MOF loading. A permeance of 1 GPU corresponds to a membrane exhibiting an intrinsic permeability of 1 Barrer and having a selective layer thickness of 1 μm. Results with * are calculated from graphs.

d Code of the different publications represented in Fig. 6.

e Optimum operation conditions that maximized gas separation performance of the MMMs. Values are given in brackets.

The first M^4^ reported^48^ comprised a three dimensional copper(ii) biphenyl dicarboxylate-triethylenediamine MOF embedded in PAET (poly(3-acetoxyethylthiophene)) and was applied in gas separation. The authors claimed that the increase in hydrophobicity of the MMMs resulted in preferential adsorption of methane, leading to an increase in CH_4_ permeability at 20 and 30 wt% of MOF loading together with a decrease in CO_2_ permeability; thus, giving rise to a reduction of the CO_2_/CH_4_ selectivity. Since this pioneering work, the field of research of MOF based MMMs has experience an exponential growth and a large number of different membranes have been reported in literature. Regarding the fillers, HKUST-1, ZIF-8 and MIL-53(Al) with and without amino group have been the most studied MOFs. As for the polymers, the organic phase used as continuous phase can be classified into low flux glassy polymers (*i.e.* PSF,^95–98^ PPEES,^99^ PVAc,^100^ Ultem®, Matrimid®^101–104^ or PBI^105–107^) and more interesting high flux polymers: rubbery, such as PDMS^96^ and PMPS (polymethylphenylsiloxane),^108^ and glassy, such as 6FDA-DAM.^40,109^

As a general trend, in a large percentage of the reported results, improvements in flux at constant selectivities with respect to the bare polymer have been reported and only in circa 10% of the cases improvements in both flux and selectivity were achieved. Furthermore, for all the membranes tested at high pressures it was observed that upon MOF addition, the plasticization of the membrane at high CO_2_ pressures was partially suppressed, maintaining large separation factors over a wider pressure range than that observed for the pure polymer^135,157^ or even increasing the selectivity at high pressures.^45,143^

This behaviour has very important consequences in applications in which the retentate has to be kept pressurized. These improvements in permeability and/or selectivity upon filler addition demonstrate the tremendous potential of MOF-based MMMs for efficient CO_2_ capture, as can be seen when results are put in perspective using the customary Robeson plots with the latest upper limits (see Fig. 6).^23^

For CO_2_/CH_4_ separation, membranes comprising high permeable 6FDA-containing polyimides (*e.g.* 6FDA-4MPD,^40,109^ 6FDA-ODA^123,162^ and 6FDA-DSDA^163^) have generally a performance beyond the Robeson limit of 1991,^164^ surpassing the Robeson limit of 2008^23^ when ZIF-90^40^ and [Zn_2_(1,4-bdc)_2_(dabco)]·4DMF·0.5H_2_O are used (graph codes 11C and 2B, respectively). Furthermore, for this gas mixture M^4^ based on a microporous polymer PIM-1, also exhibits a behaviour above the latest Robeson limit, reaching permeabilities up to 6300 Barrer together with selectivities of 14.2 for ZIF-8 loadings of 39 wt%.^126^ However, as expected, low permeable polymers, such as PSF, Ultem®, PPEES or Matrimid® lead to composites with separation properties well below the state of the art, with permeabilities typically in the range 2 to 70 Barrer and selectivities between 8 and 135. In the case of CO_2_/N_2_ separation, the best results have been obtained for Pebax® and PIM-1 whose permselectivities have been improved up to above the latest Robeson limit upon ZIF-7 and ZIF-8 addition. Interestingly, M^4^ comprising Pebax® and 35 wt% ZIF-8^139^ and 6FDA-DAM with 10 wt% CPO-27(Mg)^137^ have attractive separation properties for the separation of CO_2_ from flue gas on a large scale when cross-flow modules with membranes with selective layers thinner than 300 nm operated at pressure ratios of 5–10 are considered.^82^

Moreover, membranes comprising ZIF-8 and 6FDA-DAM:DABA, PIM-1 or 6FDA-durene (graph codes 24, 27 and 42 respectively),^126^ CPO-27 and XLPEO (graph code 37A),^137^ and [Zn_2_(1,4-bdc)_2_(dabco)]·4DMF·0.5H_2_O and 6FDA-4MPD (graph code 2B) exhibit permselectivities very close to those required for an attractive membrane-based post-combustion CO_2_/N_2_ separation.^82^

However, it must be highlighted that the optimal membrane performance calculated by Merkel *et al.*^82^ concerns flue gases consisting of low CO_2_ concentrations in N_2_ at 40–50 °C saturated in water. In this sense, more realistic measurements of these membranes, including water vapour, should be performed to assess their viability. Finally, for H_2_/CO_2_ separation, membranes comprising PBI,^105^ PIM-1^126^ and 6FDA^114^ containing polyimides and ZIFs (namely, ZIF-8,^107^ ZIF-90,^128^ ZIF-7^105^ and ZIF-11^158^) exhibit the best performance. The most outstanding results were obtained for asymmetric membranes prepared with HKUST-1 and PMDA-ODA^30^ and dense membranes containing ZIF-8 and PBI^107^ for which the commercial attractive region^89^ is reached with MOF loadings of 6 wt% and 30 wt%, respectively. Interestingly, the membranes were tested up to 230 °C in the latter case, under conditions relevant for pre-combustion CCS.

## E Towards high productivity M^4^s: progress in hollow fiber and thin layer membranes

A membrane module with a proper permselectivity for a real industrial application should also have a meaningful productivity. Even membrane materials displaying excellent separation performance would be useless if productivity is low. By controlling the morphology it is possible to create asymmetric (anisotropic) membranes with very thin selective layers that decrease mass transfer resistance and increase productivity.

Generally the target is to have a selective layer with a thickness lower than a micrometer. However, such a thin layer of polymeric or mixed matrix material needs a support. This is the basic definition of asymmetric membranes: a thin selective dense layer on a non-selective porous support providing the strength. Several methods to manufacture asymmetric membranes are available, such as phase separation, interfacial polymerization, solution-coating, plasma polymerization, *etc.*^165^

**Fig. 7:**
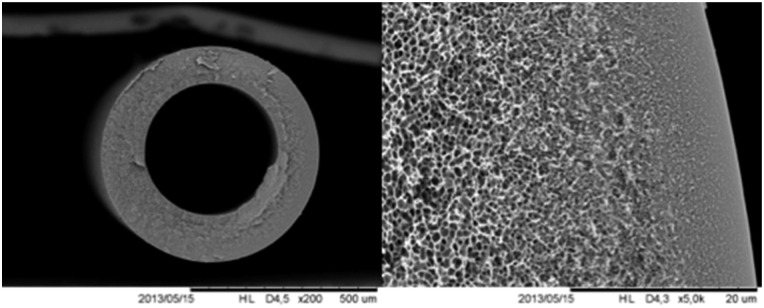
SEM images of the cross section of a polysulfone hollow fiber membrane, (a) overall, (b) outer edge.

A fundamental question when it comes to the application of thin separating layers regards their geometry. Basically, two different membrane modules can be envisaged: (i) spiral wound flat sheets, (ii) supported composites and (iii) hollow fiber (HFb, see Fig. 7 membrane modules). Although spiral wound modules were the first commercialized, HFb modules offer significant advantages; the most important being their high packing density (over 10 000 m^2^ m^−3^),^166–168^ about ten times higher than for flat sheet (plate and frame) membranes. In addition, HFb membranes can handle very high transmembrane pressure differences (up to 70 bar) and their fabrication costs are 5 to 20 times lower than that of equivalent membranes for spiral wound modules (5–20 US $ per m^2^*versus* 5–100 US $ per m^2^).^158^ Although already some reports exist on M^4^ based asymmetric flat membranes,^113,116,120,125,169,170^ with promising results and providing important insight, because of the above reasons we focus here on asymmetric hollow fiber membranes.

The preparation of hollow fiber membranes relies on the phase separation technique developed by Loeb and Sourirajan,^165,171^ most specifically, phase inversion spinning (also called dry jet-wet quench spinning or wet spinning).^167,168^ In this process, a dense layer is integrally formed over a porous layer. The outer dense thin layer (selective skin layer) is the selective part of the structure while the inner porous layer is only a support without any important transport resistance. In the process, a polymer solution and a bore fluid are coextruded through a nozzle (spinneret) and precipitated in a non-solvent bath to create the asymmetric structure. Thickness of the selective layer and the morphology of the substructure determine the efficiency of the membrane.

Besides the parameters to control for the fabrication of polymer-only fibers, spinning mixed matrix membranes in asymmetric hollow fiber geometry (MM-HFbM) brings a few more issues to address, namely the compatibility of the polymer and filler particles and the distribution of the particles within the fiber wall. Zhang and co-workers^146^ defined the preparation of MM-HFbM as the development of an asymmetric structure from compatible components (polymer and filler) with a very thin selective layer where the filler particles are well dispersed without any major defect at an economically attractive cost.

The first key parameter to achieve this objective is the preparation of a homogenous dispersion of the particles within the spinning solution, since stability of the dope during the spinning process is crucial to avoid sedimentation or cluster formation. Studies with polymer–zeolite dense films and MM-HFbMs showed that surface interactions between filler particles and other components of the polymer solution (‘dope’) is the parameter to tune in order to increase the stability and the performance of the membranes. This is mostly discussed as polymer–filler interaction or compatibility and becomes even more important in the case of sub-micron particles.^146,167^ The controlling parameters are: (a) the surface properties of filler particles, such as the degree of hydrophobicity, (b) surface chemistry, (c) surface charge, (d) geometry and (e) the size of the particles. It is also known that the nature and degree of interaction of the (non)-solvents with filler particles are important parameters that control the phase separation kinetics.^73^

The distribution of filler particles within the polymer matrix in the thin separating layer is the other key issue that affects performance of a MM-HFbM. There are different methods for dispersing particles during the dope preparation process.^146,166,167^ Mostly, particles are wetted and dispersed in a small portion of the solvent of the dope formulation. Then this dispersion is added to the dope solution or *vice versa*. The energy required for dispersing is introduced by mechanical agitation or sonication. Mechanical agitation is generally performed by high speed mixers. Ultrasound baths (indirect sonication) or horns (direct sonication) are used when mechanical agitation is not enough. Indeed, indirect sonication seems to be the most efficient method to avoid the formation of agglomerates.^121^

Even if a good dispersion is obtained, agglomeration and settling of the particles before spinning could be a problem. This issue may be controlled by tuning the flow behaviour of the dope. It has also been observed that agglomeration of particles during the phase inversion is possible depending on the concentration of the particles within the dope.^166^ Agglomeration of particles can cause serious defects within the outer dense selective layer and ends with performance loss. Distribution, compatibility and agglomeration issues should be considered together. Goh *et al.* showed that the agglomeration of filler particles and gap formation at the filler–polymer interface are strongly related.^172^ They observed that suppressing the agglomeration yields fewer defects at the selective surface. Also they claim that the formation of large voids with tear-drop shape in the sub-structure is strongly related to agglomeration phenomenon. Spinning parameters, related to the flow behaviour – momentum and velocity profiles at the outlet of the spinneret nozzle – have a great influence on the orientation of the polymer chains and the redistribution of particles along the fiber wall.^173–178^ There are two sources of stress induced on dope and/or nascent fiber during the spinning process; the shear stress induced within the spinneret and the stress on the free falling (or pulled by a take-up drum) nascent fiber from the spinneret nozzle. These stresses change the alignment and the orientation of polymer chains and filler particles. On the contrary, they also may have a negative effect on the adhesion of the polymer on the surface of the fillers.^167,179^

Last but not least, selectivity of an asymmetric MM-HFbM is very sensitive to the state of the selective layer. There is a strong relation between the degree of defectiveness and thickness of the selective layer.^168^ In order to overcome the formation of defects during preparation, different approaches can be followed: (i) addition of non-solvents into the dope formulation, (ii) performing the spinning at high temperatures (>50 °C) or (iii) optimizing the shear and elongational force to manipulate the polymer chain orientation and at the same time orienting the MOF particles with large aspect ratios. In addition, high filler loadings cause high viscosity that makes handling and spinning process more difficult and also can cause defects on the surface.^146^ Also, high particle loading, especially nano-size particles, is more prone to cause agglomeration. A careful attention has to be paid as well as intensive laboratory work is necessary to optimize the dope, not only to achieve good dispersion and defect free fibers, but also for handling the dope and to achieve an excellent utilization of the filler, allowing the use of lower concentrations.

Even when the issues above have been solved, almost in all cases, industrial fiber spinning yields defective membranes. The most common healing technique to allow application of these fibers consist of a thin coating with a secondary polymer (*i.e.* PDMS or Polyaramid) to clog the possible pin holes or scratches.^180,181^ This layer must be more permeable than the original selective skin to maintain productivity. The nature and the selectivity of this coating may also affect the separation characteristics of the membrane.^146^ Annealing above *T*_g_ (glass transition temperature) has also been reported as another post-treatment to heal the defects in the skin layer. Coatings by plasma polymerization and/or plasma treatment of the membrane surface are recently proposed defect healing techniques.^116^

**Fig. 8:**
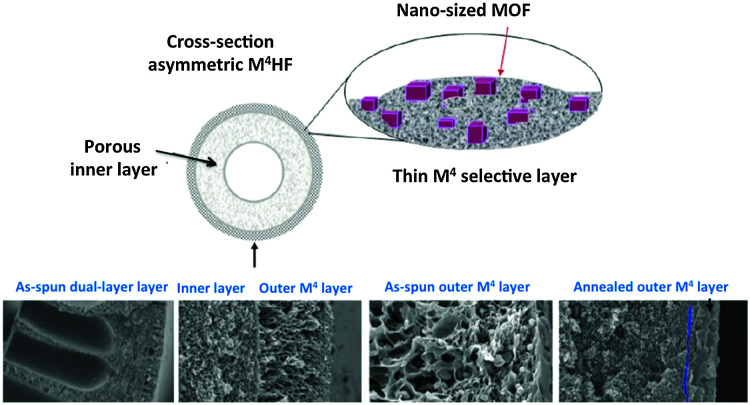
Schematic cross-section morphology of the dual-layer hollow fiber with a polymer–particle mixed matrix skin.^168^ Reproduced with permission from Elsevier.

The dual layer asymmetric hollow fiber concept represents a step forward to overcome all these issues and it offers important advantages, since this configuration allows using different polymers or different dope formulations within the same fiber. Dual layer hollow fibers are fabricated by co-extrusion of two dopes and a bore fluid, allowing the use of different polymers for support and separating layers^167–169,182^ Although this method adds further complexity, it allows the use of cheaper and unselective polymers -for the support layer. Moreover, different dope formulations can be used for the inner and outer layers.^40,168,183,184^ Another advantage is that this configuration allows the use of highly selective but not spinnable polymers (as a single layer HFb) for the skin layer. Populating the selective filler particles within the dense selective layer; dual layer fiber spinning with mixed matrix dope on the sheath and polymer only dope for the bore is an excellent alternative to control the distribution of particles (Fig. 8) and to minimize filler consumption.^168,177^ In order to avoid the formation of defects on the surface, it is necessary to use filler particles much smaller than the thickness of the selective layer.

Although most of the current experience on MM-HFbMs originates from studies on polymer–zeolite MMMs, already the first reports on M^4^-HFbs have been published. Hu and co-workers spun polyimide (PI)–Cu_3_(BTC)_2_ blends.^114^ Their results proved that particles are homogenously dispersed within the polymer matrix with only a few small agglomerates and no serious interface voids. Authors claim that the pore blocking of MOF with PI chains and the rigidification of PI chains within the interface negatively affect the diffusion of all gases, reaching a maximum permeance for H_2_ of 1270 GPU with a selectivity over CO_2_ of 27 (already within the desired range for pre-combustion CO_2_ capture). In a more recent study, Dai *et al.* prepared dual layer M^4^-HFbs with a commercial polyetherimide (Ultem®100) and ZIF-8 fillers for CO_2_/N_2_ separation.^119^ Morphological characterization studies showed a homogeneous dispersion of the filler material (size ∼ 200 nm) and a very good adhesion between the core and the sheath regions. Gas permeation measurements demonstrated that both permeance and permselectivity of dual-layer MM-HFbMs are higher than that of polymer only HFb membranes. At similar test conditions, Ultem®-ZIF-8 MM-HFbMs yielded a CO_2_/N_2_ selectivity (= 36) with relatively low permeance for CO_2_ (= 26 GPU). The actual challenge is to decrease the skin thickness to achieve higher fluxes. In the latest report on the subject, researchers reported the successful formation of high-loading mixed matrix hollow fibers containing ZIF-8 (up to 30% wt).^146^ Although the study targeted hydrocarbon separations, it proves that it is feasible to transfer the knowledge generated from dense film studies on MOF containing mixed matrix membranes to industrial scale highly productive asymmetric hollow fiber membranes.

## F Structure performance relationships in M^4^s

As thoroughly discussed above, polymer as well as filler properties affect MMM morphology and separation performance. Regarding the filler, chemical structure, surface chemistry, particle size distribution and aspect ratio are the most important variables. Indeed, poor filler–polymer adhesion and filler segregation or blocking of its porosity by the polymer are the main reasons why traditional MMM fillers like zeolites, silicas or activated carbons have not made the final steps towards industrial implementation. Due to these limitations, in general only low filler loadings can be achieved without compromising the separation performance unless laborious filler post-treatments are applied.^157^ As to the polymer, it is very important to match its properties with those of the MOF filler.^4^

For every MOF–polymer couple, the MOF loading should be maximized. Loadings lower than a certain value do not alter in a significant way the transport properties of the polymer membrane, while rigidity and mechanical strength of the composite are increased, as determined by differential scanning calorimetry and dynamic mechanical analysis.^37,101,115,185,186^ At a certain loading, a good dispersion of the filler with an excellent interfacial contact with the polymer chains (composite interface) results in an optimum MMM performance. However, at higher loadings polymer chains are not completely able to enwrap the particles, so that the latter may agglomerate, reducing their dispersion in the polymer matrix and forming undesirable transport channels.^96,103^ High permeabilities are also attributed to the disruption of the polymer chain packing and linking due to the presence of the molecular sieves which implies also an increase in polymer free volume.^187^ In this section, we first discuss how to assess the exact structure of these composites, then the different synthetic approaches utilized to improve MOF–polymer performance. Finally, we will introduce the use of Hansen solubility parameters to predict chemical interactions between MOF and polymer.

Structural features of MMMs such as the spatial distribution of the filler crystals and the existence of voids at the filler-matrix boundary are essential in determining the mechanical properties and gas separation performance. However, these parameters are also particularly difficult to assess experimentally. Generally, the membranes are fractured after immersion in liquid N_2_, often referred to as cryo-fracturing, in order to gain access to the cross-section of the membrane with imaging techniques such as SEM. This provides only 2D, local information, while furthermore the cryo-fracturing approach often results in rough membrane cross-section surfaces. Therefore, a number of surface motifs derived from the fracturing process are imaged, leading to an incomplete or deceptive picture of the structural features of the membrane, most particularly the fraction of voids in the membrane.

**Fig. 9:**
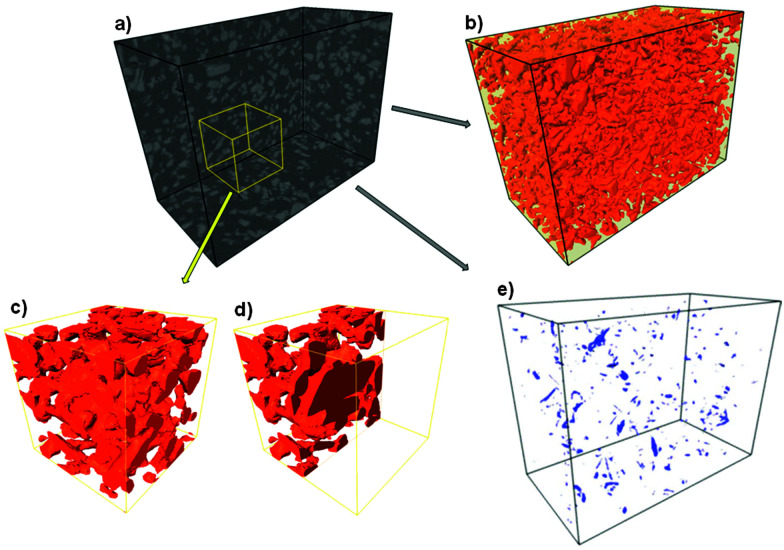
3D reconstructed volume of the portion of a NH_2_-MIL-53(Al)/PI_25% membrane studied with FIB-SEM (a); the corresponding surface-rendered view of the volume corresponding to the MOF crystals (b). Panel (c) shows a detail of the volume indicated in the yellow frame in panel (a). In panel (d), half of the material in (c) has been removed to improve visualization. And (e) shows the surface-rendered view of the volume corresponding to the voids. Box size (a, b, e): 14.3 μm × 10.7 μm × 7.5 μm.^46^ Reproduced with permission from WILEY-VCH Verlag GmbH & Co.

As an interesting alternative, the use of FIB-SEM tomography^188^ in the characterization of M^4^s was recently introduced by Rodenas *et al.*^46,189^ In FIB-SEM imaging the Ga^+^ primary focused ion beam is used to controllably sputter a selected area of the specimen, precisely removing thin slices of material and enabling a series of consecutive cross-sections to be studied individually. Here FIB-SEM was used to study the spatial distribution of the MOF filler in the MMMs as well as a mean to quantify the contact between the filler and the matrix phases, as depicted in Fig. 9. After alignment of the stack of SEM micrographs, the 3D structure of the analysed volume could be reconstructed in 3D and depicted along three orthogonal cross-sections. Segmentation of the individual phases, *i.e.* PI matrix, MOF filler and voids, was performed by image thresholding. Despite the relatively high filler loading (25 wt%), a homogeneous distribution of the MOF crystals within the polymer matrix is observed, indicating adequacy of the procedure employed to cast the MMMs. In addition, quantification of the segmented volume allowed determining the mass based MOF loading, obtaining a remarkably good agreement with the bulk MOF loading (as derived from TGA), which validates the image analysis results. With this new technique, it is now possible to have a much more realistic picture of the internal structure of the membrane. In combination with rigorous mathematical modelling, as the one developed by Nair and co-workers, a unique tool for the understanding of MMMs should be available for future studies.

One of the most critical concerns for the development of MMMs is the lack of compatibility between filler and polymer matrix, which produces a decrease in membrane performance. To overcome this issue, different strategies have been developed in the last years. An elegant solution is the use of MOFs that contain organic linkers similar to elements of polymer units. In particular, ZIFs have been regarded as promising fillers for MMMs because the linkers in ZIF structure are expected to allow a better affinity and interaction with certain polymeric materials such as polybenzimidazole (PBI). Yang *et al.*^105^ showed that it is possible to prepare M^4^s containing up to 50 wt% of ZIF-7 nanoparticles by mixing the as-synthesized ZIF-7 nanoparticles without the traditional drying process with PBI. In these M^4^s the permeability of H_2_ was significantly higher than in pure PBI membranes, although H_2_/CO_2_ selectivity was not improved. In a similar way, M^4^s containing PBI and high loadings of ZIF-8 or ZIF-90 nanocrystals^106,128,190^ have been fabricated. As expected, all these composite membranes showed improved H_2_ permeability together with an enhanced H_2_/CO_2_ separation selectivity.^128^ Recently, Yao *et al.*^158^ fabricated M^4^s incorporating ZIF-11 crystals into PBI. Gas permeabilities of H_2_ and CO_2_ increased upon incorporation of ZIF-11. Additionally, the H_2_/CO_2_ ideal selectivity was also slightly improved in the composite membrane containing a 16 wt.% of MOF.

MOFs containing amino groups are also considered good candidates to optimize polymer matrix-filler interactions. In particular, the flexible NH_2_-MIL-53(Al) has shown excellent adhesion with different polymers such as polysulfone or polyimide.^45,162^ Nik *et al.*^123^ reported that fillers containing amino groups may lead to the rigidification of the polymer at the MOF–filler interface, thus decreasing gas permeability and increasing selectivity. However, almost no systematic study about the influence of functional groups on the MOF–polymer interactions has been performed. Seoane *et al.*^163^ synthesized thioether- and sulfone-containing copolyimides, 6FDA:DSDA/4MPD:4,4′-SDA (polymer 1) and 6FDA/4MPD:4,4′-SDA (polymer 2), with the aim of studying the effect of polymer functional groups in the preparation and performance of M^4^s containing NH_2_-MIL-53(Al) or NH_2_-MIL-101(Al). The main difference between both polymers was that in polymer 1 a part of the 6FDA monomer was substituted by the more flexible DSDA unit. This modification increased the interaction between the amino-functionalized MOFs and polymer 1, showing that the flexibility of the polymer had an influence on the filler–polymer matrix interaction and, consequently, on the overall performance of the membranes.

MOF–polymer interactions can also be improved using the one-pot synthesis methodology developed by Seoane *et al.*^131^ A common solvent for the MOF synthesis and membrane casting is necessary in this approach.

Other methodologies have been used to improve the MOF distribution in the polymer matrix. Interfacial polymerization of thin film nanocomposite membranes was first developed by Jeong *et al.*,^191^ and allows embedding the filler nanoparticles into a thin polymer film during monomer polymerization. Sorribas *et al.*^192^ have used this procedure to obtain MOF [ZIF-8, MIL-53(Al), NH_2_-MIL-53(Al) and MIL-101(Cr)]-polyamide (PA) thin layers on top of cross-linked polyimide porous supports. The use of fillers of different nature within the same composite is another interesting approach towards improved separation performance, as introduced by Zornoza *et al.*^97^ in a study where polysulfone based MMMs containing one MOF (HKUST-1 or ZIF-8) and zeolite silicalite-1 were manufactured. Later Valero *et al.*^145^ obtained MMMs by a combination of silica MCM-41 and MOF NH_2_-MIL-53(Al) in glassy commercial polymers (polysulfone Udel® or polyimide Matrimid®) following a similar approach.

The so-called Hansen solubility parameters (HSP) have classically been applied to the evaluation of solvent-polymer chemical interactions,^193^ but also to study barrier properties and chemical resistance of protective clothing,^193^ prediction of cytotoxic drug interactions with DNA,^194^ optimization of the extraction of bioactive compounds from biomass with subcritical water,^195^ identification of an alternative, less toxic solvent used in a microencapsulation process,^196^ and preparation of stable dispersions of TiO_2_ and hydroxyapatite nanoparticles in organic solvents,^197^ among other examples. In the case of polymeric membranes, these parameters have been used to evaluate possible solvents and non-solvents to prepare membranes by phase inversion,^198,199^ to study membrane fouling,^200^ for the analysis of compatibility between polymers and IPA in IPA/water distillation^201^ and to explain the acetone, butanol and ethanol interaction with silicalite-1/PDMS membranes.^202^

Regarding the application of HSP to metal–organic framework (MOF) materials, there are two recent reports; one dealing with the formation of composites between MOF HKUST-1 (with 5 wt% loading) and poly(l-lactic acid) (PLLA),^203^ and other discussing the encapsulation of caffeine into MOFs ZIF-8 and NH_2_-MIL-88B.^204^ Thanks to HSP the basic principle of “like dissolves like”, *i.e.* the qualitative idea behind most of the previous examples, is expressed in numbers easy to handle and compare. However, a limitation of HSP application is related to insufficient availability of HSP data for systems of interest (in particular, MMM polymers and mainly MOFs).

The chemical interactions established between MOF and solvent or ligand can be of different nature, *i.e.* dispersion, polar or hydrogen bonds. In case of solvents these interactions can be discussed in terms of Hansen solubility parameters.^193^ These parameters (*δ*_D_, *δ*_P_ and *δ*_H_ for dispersion or London interaction, polar interaction and hydrogen bonds, respectively) are given in Table 1 for some selected membrane polymers, MOF ligands and MOF HKUST-1 (the only available MOF to date with HSP^203^). The MOF ligands included in this table are commonly used for the synthesis of some of the most typical and studied MOFs: 2-methylimidazolate (2MI, for ZIF-8^205^), benzenedicarboxylate (BDC, used for MOF-5,^206^ MIL-53,^207^ MIL-101,^208^ UiO-66,^209^*etc.*), NH_2_-benzenedicarboxylate (NH_2_-BDC, used for NH_2_-MIL-53,^210^ NH_2_-MIL-88B,^211^*etc.*) and benzenetricarboxylate (BTC, for HKUST-1,^212^ MIL-96,^213^*etc.*). In terms of HSP, the interaction between two substances 1 and 2 can be obtained calculating the parameter *R*_a_^193^ with the following eqn (1):

17 *R*_a_^2^ = 4(*δ*_D1_ − *δ*_D2_)^2^ + (*δ*_P1_ − *δ*_P2_)^2^ + (*δ*_H1_ − *δ*_H2_)^2^

In our case *δ*_D1_, *δ*_P1_ and *δ*_H1_ and *δ*_D2_, *δ*_P2_ and *δ*_H2_ sets of parameters would correspond to polymer and MOF ligand, respectively. Since HSP are not available for MOFs (with the exception of HKUST-1^203^) we have simplified the approach for the forthcoming discussion by attributing to the ligand the solubility properties of the MOF, or equivalently by making the assumption that the linker–polymer interactions dominate the MOF–polymer interactions. This approach is similar to that assumed by Hansen when used HSP of DNA base segments to estimate affinity between cytotoxic drugs and DNA itself,^194^ and by Paseta *et al.*^204^ when HSP is used to discuss the encapsulation of caffeine into ZIF-8 and NH_2_-MIL-88B. Although “large” HSP distances imply poorer interaction, specifically, in case of polymer-solvent, *R*_a_ values below about 7.5 meet the Flory–Huggins criterion for compatibility^214^ which at least gives us a starting scale for looking at MOF–polymer *R*_a_ values.

*R*_a_ values in Table 3 suggest that common linkers are not totally compatible with the selected membrane polymers (*R*_a_ above 7.5); however, 2MI (in special) and BDC are compatible with four out of five polymers (excluding PDMS). This is in agreement with the great availability of MMMs with MOFs obtained from 2MI and BDC (in particular the above mentioned ZIF-8 and MIL-53). When *R*_a_ values for the same polymers are compared for HKUST-1 and BTC (the linker in HKUST-1), the analysis always favours the MOF over its linker. This relative discrepancy between BTC-HKUST-1 pair (*R*_a_ in Table 1 for HKUST-1 is as good as the values commented for 2MI and BDC), is basically due to the fact that the available value for the BTC *δ*_H_ (17.0) is higher than expected because most probably all the three acid –COOH groups in BTC would not be available for H-bonding, as the experimental HKUST-1 *δ*_H_ (10.7) obtained by Auras *et al.*^203^ suggests, and as already indicated by Paseta *et al.*^204^ Furthermore, it is obvious from this discussion that the availability of HSP for MOFs of interest (so that the speculations made here for linkers would make more sense) would facilitate the selection of MOF–polymer couples without the necessity of synthesizing and testing their corresponding composite membranes. Indeed, HSP for polymers and MOFs would help selecting the best membrane polymer material for every desired MOF. Finally, it is worth emphasizing that most likely the same MOF material would exhibit different HSP values depending on the particle size (when nano- and micro-sized particles of the same MOF phase would be considered) and perhaps on the form of the MOF in those cases in which flexibility is a key issue.^207^ Indeed, in addition to the chemical compatibility of polymer and filler, another important aspect in MMM performance is the morphology of the filler.

**Table 3:** Hansen solubility parameters (HSP) for some common MMM components: polymers, linkers and MOF HKUST-1. 2MI, BDC, NH_2_-BDC and BTC correspond to 2-methylimidazole, benzene-1,4-dicarboxylic acid, 2-aminobenzene-1,4-dicarboxylic acid and benzene-1,3,5-tricarboxylic acid, respectively. HSP distances between materials obtained from *R*_a_ calculations with eqn (17). In general, HSP values were obtained from literature (PES and PEI Ultem 1000 from ref. 214, PI Matrimid from ref. 198, PDMS from ref. 202, PSF Udel P-1700 from ref. 215, and HKUST-1 (CuBTC) from ref. 203) with the exception of HSPs for 2MI, BDC, NH_2_-BDC and BTC, calculated with the commercial package Hansen Solubility Parameters in Practice^216^

	HSP [MPa^0.5^]			*R*_a_ [MPa^0.5^]				
	*δ*_D_	*δ*_P_	*δ*_H_	2MI	BDC	NH_2_-BDC	BTC	CuBTC
PES	19.6	10.8	9.2	1.7	5.2	7.9	8.1	3.8
PEI Ultem 1000	19.6	7.6	9.0	3.6	3.9	7.8	8.3	4.4
PI Matrimid	18.7	9.6	6.7	3.2	7.1	10.6	10.8	4.3
PDMS	15.9	0.1	4.7	13.1	13.5	17.5	17.7	12.2
PSF Udel P-1700	19.0	5.9	6.1	6.0	7.1	11.2	11.7	6.5
2MI	18.8	10.7	9.7					
BDC	20.0	7.2	12.8					
NH_2_-BDC	20.8	8.6	16.4					
BTC	20.3	9.3	17.0					
CUBTC	17.9	9.9	10.7					

**Fig. 10:**
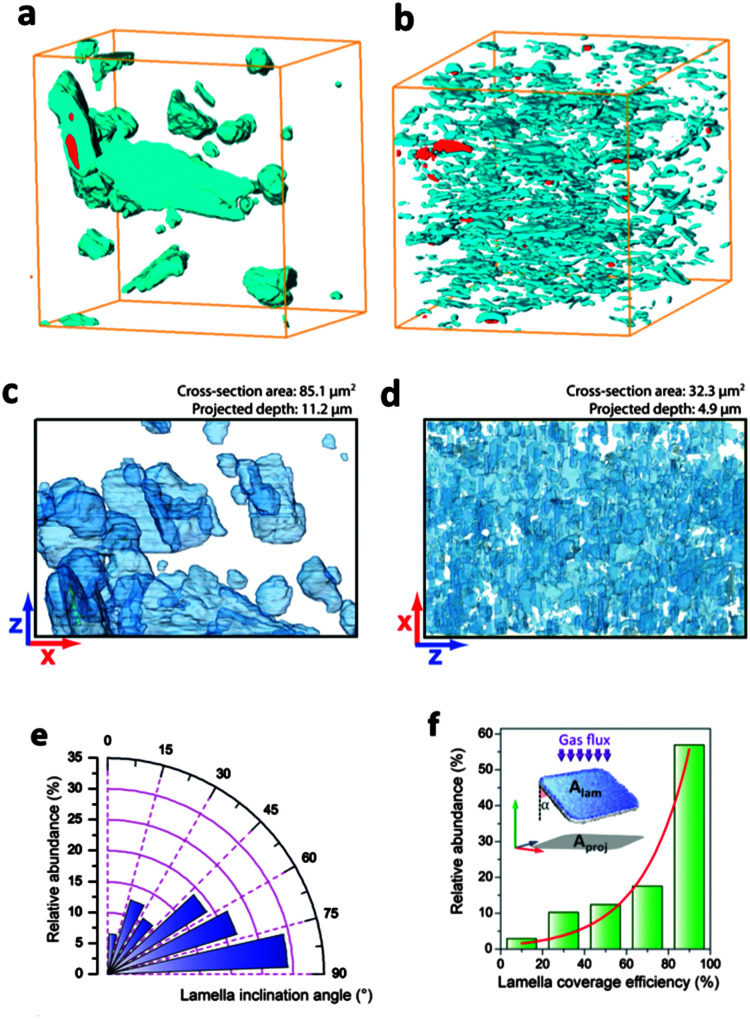
Surface-rendered views of the segmented FIB-SEM tomograms for composite membranes containing bulk-type (a) and nanosheet (b) CuBDC metal–organic-framework embedded in polyimide. Full projections along the *y*-direction of the reconstructed volumes (c, d). Angular histogram showing the orientation of MOF lamellae with respect to the gas flux direction (*y* axis) for a composite material containing MOF nanosheets embedded in polyimide (e). Histogram of the efficiency with which the individual MOF nanosheets cover the membrane cross-section, defined as the ratio between the area of the MOF lamellae (*A*_lam_) and that projected on the plane perpendicular to the gas flux (*A*_proj_), as schematically depicted in the inset to the panel (f). In the same inset figure, *α* represents the angle of inclination of each MOF lamellae with respect to the *y*-axis. Green bars correspond to experimental data while the red line shows the exponential fit. Reproduced with permission from Nature publishing group.^159^

Particle morphology is crucial for many applications and allows the properties of a certain solid to be tuned without changing the material composition. For instance, the improved performance of gold or silver nanoparticles for surface plasmon resonance,^217^ semi-conductor nanodots for quantum confinement,^218^ and metal or metal oxides for catalysis^219,220^ is strongly dependent on crystal morphology. In case of MMMs, it is easy to envisage that the performance of membranes containing exactly the same material but with a different particle configuration will result in different separation performance. For instance, most of the outstanding examples in terms of separation reported so far in literature made use of MOF nanoparticles.^40,117^ The improved performance of composites incorporating nanoparticles is usually ascribed to the larger external to internal surface ratio in these nanoparticles, that allows a much better interaction with the polymer. Moreover, lower filler loadings can be used for an improved separation performance, preserving to a large extent the mechanical properties of the polymer. However, although microwave,^221^ electrochemical^222^ protocols and the use of chemical additives^223^ are powerful synthesis tools for the manufacture of homogeneous MOF nano-crystals, conventional MOF synthesis procedures render agglomerated powders consisting of isotropic micron-sized crystals or barely dispersible nano-particles. In this spirit, the availability of high-aspect-ratio, ideally ultrathin, MOF nanostructures represents an advanced solution to improve the integration between both components in the composite materials, thereby circumventing the aforementioned hurdles. The synthesis of MOF nanosheets and their application in M^4^s has now been reported by Rodenas *et al.*^159^ In this article, a bottom-up synthesis strategy leading to highly crystalline, intact MOF nanosheets that could be readily dispersed into a polymer matrix is reported. The synthesis strategy to produce MOF nanosheets relies on the diffusion-mediated modulation of the MOF growth kinetics, with the synthesis medium consisting of three liquid layers composed of mixtures of DMF and a suitable miscible co-solvent in appropriate ratios, that are vertically arranged according to their different densities. By applying this method, the authors were able to prepare free standing nanosheets of CuBDC homologs. MOF–polymer composites were prepared by incorporating CuBDC nanosheets within a polyimide (PI) matrix at different filler loadings (2–12 wt%). The same procedure was employed to prepare comparative composites incorporating either bulk-type or sub-micron sized (nanoparticle) isotropic CuBDC MOF crystals as fillers. The internal structure of the composite membranes was again studied with FIB-SEM, as illustrated in Fig. 10. Despite the identical filler content, striking differences in the nanostructure were immediately evident. Whereas the regular MOF crystals leave a significant fraction of the composite volume unoccupied in b-CuBDC(8)@PI, due to their bulky nature, the MOF lamellae are uniformly distributed over the inspected volume for ns-CuBDC(8)@PI. Image analysis of the FIB-SEM tomograms allowed quantification of a number of structural parameters of the composite membranes (Fig. 10). The MOF nanosheets in ns-CuBDC(8)@PI exposed *ca.* one order of magnitude larger surface area than the bulk-type crystals incorporated to b-CuBDC(8)@PI (2.2 × 10^−3^*vs.* 2.9 × 10^−4^ nm^2^ nm^−3^ MOF), enormously increasing their interaction with gas molecules. As a result, at every studied *trans*-membrane pressure difference, the separation selectivity for the nanosheet-CuBDC(8)@PI membranes is 30–80% higher than for the polymeric membrane and 75% to 8-fold higher than for the bulk-CuBDC(8)@PI counterpart in the range of operation conditions investigated. The similar intrinsic sorption properties of bulk-type and nanosheet CuBDC crystals cannot account for such remarkable differences in separation performance, which were therefore attributed to the different MOF crystal morphology, which turned out key for the filler–polymer integration and the occupation of the gas permeation pathways by the molecular sieve. Most remarkably, the selectivity achieved with ns-CuBDC(8)@PI was retained or even increased slightly upon increasing the upstream pressure. This significant finding is completely opposite to the general observation for both polymeric and conventional MOF–polymer membranes,^55^ that the separation selectivity decreases with increasing partial pressure of CO_2_. These results confirm the relevance for the separation performance of the extent to which the MOF filler occupies the membrane cross-section perpendicular to the gas flux and the importance of crystal engineering in the development of efficient composites for gas separation. Moreover, while based on the Maxwell model for MMMs with homogeneously dispersed fillers an optimal performance requires similar permeabilities of both components, using fillers with large aspect ratios this requirement is strongly relaxed.^60,224^

## G Summary and future perspectives

Metal–organic framework–polymer composites hold great promise for application as gas selective membranes. In this review we have shown that already reported M^4^s, if applied in pre and post-combustion CO_2_ capture during energy generation, may be able to facilitate the development of efficient and economically affordable capture technologies. The success of these new membranes lies in the rich chemistry behind MOF formation, both in terms of chemical composition and of particle morphology. The combination of these features with highly permeable polymers can, in principle, deliver membranes that meet the most important requirements for the capture of CO_2_ under relevant post-combustion conditions and of H_2_ under relevant pre-combustion conditions. Having said this, one should also realize that the final success of these membranes will depend on several critical issues, *viz.* the manufacture of thin membranes and the closely related structure–performance relationship of M^4^s, as discussed below.

One of the most important aspects for the final application of M^4^s will be the development of efficient methods for the synthesis of thin separating layers, preferably with hollow fiber architecture. Although already some work has been devoted to this important field, much more is necessary. One of the main barriers to optimize HFb manufacture is the scaling up of state of the art MOF fillers and polymers, since large amounts of both materials are needed in regular spinning setups. In this sense, alternative thin film preparation techniques may facilitate the development of membranes with this morphology on a more controllable manner. The Langmuir–Blodgett (LB) method is a well-known technique for the fabrication of monolayer films at the air–water interface. Moreover, these films can be transferred onto almost any desired substrate to obtain films with a controlled thickness by consecutive depositions.^225^ Therefore, it is a powerful tool for the fabrication of very thin selective layers onto porous supports for the development of asymmetric membranes such as hollow fibers. Many studies dealing with the use of LB films for gas separations resulted in disappointing results, since the selectivities obtained were lower than the values predicted from Graham's law. This indicated that gas transport occurred mainly through film defects.^226^ To improve the selectivity of LB films in gas separations, the group of Regen started in the early 1990's a systematic study to obtain defect-free LB films making use of different surfactants based on the calix[6]arene framework.^227^ In a recent publication,^228^ the authors have shown that an asymmetric membrane formed by a 7 nm thick bilayer composed by a quaternary ammonium derivative of poly(maleic anhydride-*alt*-1-octadecene) ionically cross-linked with poly(acrylic acid) deposited onto a cast film made from poly[1-(trimethylsilyl)-1-propyne] (PTMSP) exhibits a remarkable H_2_/CO_2_ selectivity of 200 (with a H_2_ permeability of about 9 GPUs). These contributions have shown that it is possible to obtain very thin dense layers that are suitable for the development of gas separation membranes by the LB technique. Furthermore, Tsolatas *et al.*^229^ obtained two-dimensional MOF LB films using three kinds of MOF particles with different sizes and morphologies, Cu_3_(BTC)_2_, Cu_2_(BDC)_2_(BPY) and Al_12_O(OH)_18_(H_2_O)_3_(Al_2_(OH)_4_)(btc)_6_. These authors showed that the morphology of the MOF crystals determines the particle orientation on the substrates; moreover, crystal density on the films could be controlled with the LB method. Additionally, Lu *et al.*^230^ have obtained monolayers of polyvinylpyrrolidone-modified UiO-66 microcrystals at the air-liquid interface using sodium dodecyl sulfate to consolidate the films. Hybrid films MOF–polymer obtained by the LB method would allow a deeper understanding of the filler–polymer interactions and also a more detailed characterization of the membrane structure and properties and of the subsequent influence on the MMM performance. Different strategies may be used to obtain mixed MOF–polymer LB films. Xu and Goedel^231^ produced polymer–silica hybrid LB films spreading a chloroform solution containing hydrophobized silica colloids (140 nm of diameter) and polyisoprene amphiphiles (47/53 wt%) onto a water surface. After compression, the hybrid monolayer was cross-linked by UV light and transferred onto different solid supports to obtain a freestanding cross-linked hybrid membrane. The fabrication of hybrid monolayers polymer/nanospheres at the air–water interface has been reported by Hu *et al.*^232^ The authors spread polystyrene spheres with diameters ranging from 100 nm to 1 μm onto the water (containing 1–3 ppm of polyethylene oxide) surface. In a few hours, the polymer was adsorbed onto the surface of the nanospheres obtaining a closed packed hybrid monolayer that could be transferred onto a solid support. Recently, Martin-Garcia and Velazquez^233^ obtained hybrid LB films composed by CdSe quantum dots (3.5 nm) and poly(styrene-*co*-maleic anhydride) partial 2-butoxyethyl ester cumene terminated (PS-MA-BEE) by successive compression–expansion cycles^234^ of the monolayer at the air–water interface. This study has shown that the morphology of the hybrid films can be modulated by shear stress. In addition, the LB method can be used to fabricate alternate LB films by successive deposition of monolayers of different materials, what would allow obtaining polymer–MOF–polymer sandwich-like structures with a controlled density of MOF particles. All these strategies and many others that can be proposed for the fabrication of mixed MOF–polymer LB films open an attractive field of research for the next years. On a different type of approach, the preparation of pure MOF coatings on polymeric HFb supports by interfacial microfluidic processing has also attracted considerable attention in the last few years, with very promising results.^235–239^

In spite of the preparation method, it is very important to gain much more insight into the relationship between composite structure, components and membrane performance. The development of accurate mathematical models to describe transport among M^4^s, the use of adequate techniques for the characterization of these membranes and the appropriate selection of components are of the utmost importance. Preparation and testing of M^4^s should not rely on serendipity but on the judicious choice of components. In this sense, the development and use of indicators like the Hansen solubility parameters may offer great advantages in the future and should help researchers make a first selection of components rather than trying every MOF and polymer “from the self”.

In this review we have highlighted the importance of crystal engineering as a powerful tool to further enhance membrane performance. Fillers with large aspect ratios and/or in nanoparticulate form seem to be the most feasible ones to achieve improved separation performance and thin films, the latter being a must to achieve the necessary productivities for pre- and post-combustion CO_2_ capture.

Last but not least, separation performance under conditions relevant for practice (*e.g.* long-term operation at high temperature and/or pressure with realistic multicomponent mixtures) will be necessary to convince industry about the applicability of M^4^s. MOFs are still seen as unstable materials by a large part of the scientific community and by most industry. Although M^4^ literature points at a very beneficial effect of the polymer matrix into MOF stability, more proofs and long term testing under real separation conditions (both in terms of gas composition and temperature) will be required.

In summary, M^4^s are at the forefront of MOF and membrane research and the next few years will be crucial in the future of these thrilling composites. We are however confident that even more exciting results will be achieved by the scientific community and these should pave the way to the, perhaps, first large scale application of metal–organic frameworks.

## Supplementary Material

CS-044-C4CS00437J-s001
