# Supplementary material for: Metal–organic framework based mixed matrix membranes: a solution for highly efficient CO2 capture?
Source: Chem Soc Rev. 2015 Feb 18;44(8):2421–54. doi: 10.1039/c4cs00437j (PMC4445399; doi:10.1039/c4cs00437j)
Supplement: CS-044-C4CS00437J-s001 [file CS-044-C4CS00437J-s001.pdf]

Electronic Supplementary Material (ESI) for Chemical Society Reviews.
This journal is © The Royal Society of Chemistry 2015

Supplementary Information to

Metal Organic Framework based Mixed Matrix Membranes: a solution for highly efficient CO_2 capture?

Beatriz Seoane[1], Joaquin Coronas[2a], Ignacio Gascon[2b], Miren Etxeberria Benavides[3], Oğuz Karvan[3], Jürgen Caro[4], Freek Kapteijn[1] and Jorge Gascon[1,*]

[1] Catalysis Engineering, ChemE, Delft University of Technology, Julianalaan 136, 2628 BL Delft, The Netherlands.

[2a] Chemical and Environmental Engineering Department and Instituto de Nanociencia de Aragón (INA), Universidad de Zaragoza, 50018 Zaragoza, Spain.

[2b] Physical Chemistry Department and Instituto de Nanociencia de Aragón (INA), Universidad de Zaragoza, 50018 Zaragoza, Spain.

[3] Tecnalia, Energy and Environmental Division, Parque Tecnológico de San Sebastián, Mikeletegi Pasealekua 2, E-20009 Donostia-San Sebastián, Spain.

[4] Institut für Physikalische Chemie und Elektrochemie, Leibniz Universität Hannover, Callinstr. 22, D-30167 Hannover, Germany.

[*]Correspondence to: j.gascon@tudelft.nl

List of acronyms and abbreviations

μ-BPA	1,2-Bis(4-pyridyl)ethane
μ-BPP	1,3-Bis(4-pyridyl)propane
1,4-BDC (or BDC)	1,4-Benzenedicarboxylate
2,6-NDC	2,6-Napthalenedicarboxylate
2-amBzIM	2-Aminobenzimidazole
2MI	2-Methylimidazole
4,4' BPDC	4,4'-Biphenyl dicarboxylate
4,4'-BPY	4,4'-Bipyridine
4,4'-SDA	Bis(4-aminophenyl) sulphide
4MPD	2,3,5,6-Tetramethyl-1,4-phenylenediamine
6FDA	4,4′-(Hexafluoroisopropylidene)diphthalic anhydride
6F-PBI	Hexafluoroisopropylidene-containing polybenzimidazole
APTMDS	Bis(3-aminopropyl)tetramethyldisiloxane
B	Bulk crystals
BTC	1,3,5-Benzenetricarboxylate
BuI	5-Tert-butylisophthalic acid
BzIM	Benzimidazole
CCS	Carbon Capture and Storage
CLC	Chemical looping combustion
CMS	Carbon molecular sieves
DABA	3,5-Diaminobenzoic acid
DABCO	1,4-Diazabicyclo[2.2.2]octane
DAM (or TMPDA)	2,4,6-Trimethyl-*m*-phenylenediamine
DBzPBI	PBI after N-substitution reaction with 4-tert-butylbenzyl bromide
DEA	Diethanolamine
DMF	Dimethylformamide
DMPBI	PBI after N substitution reaction with methyl iodide
DSDA	3,3',4,4'-Diphenylsulfone tetracarboxylic dianydride
ETS	Emissions Trading System
Glu	Glutarate
GPU	Gas permeance units
H_2hfipbb	4,4'-Hexafluoroisopropylidene bis(benzoic acid)
HFb	Hollow fiber
HFS	Hexafluorosilicate
HHV	Higher heating value
HSP	Hansen solubility parameters
IAST	Ideal adsorbed solution theory
IGCC	Integrated gasification combined cycle
IPA	Isopropanol
IPCC	Intergovernmental Panel on Climate Change
LANL	Los Alamos National Laboratory

LB	Langmuir-Blodgett
LCOE	Levelized Cost of Energy
M^4	MOF-based mixed matrix membrane
M^4-HFb	MOF-based mixed matrix membrane in asymmetric hollow fiber geometry
MEA	Monoethanolamine
MM-HFbM	Mixed matrix membranes in asymmetric hollow fiber geometry
MMM	Mixed-matrix membrane
MOF	Metal organic framework
MS	Maxwell-Stefan
MSS	Mesoporous silica spheres
MTR	Membrane Technology and Research, Inc.
Nc	Nanoparticle crystals
NH_2-BDC	2-Aminoterephthalate
Ns	Nanosheets
ODA	4,4'-Oxydianiline
ODPA	4,4’-Oxydiphthalicanhydride
PA	Polyamide
PAET	Poly(3-acetoxyethylthiophene)
PAI	Polyamide-imide
PBI	Polybenzimidazole
PC	Pulverized coal
PDMS	Polydimethylsiloxane
PEI	Polyethylenimine
PES	Polyethersulfone
PI	Polyimide
PIM	Polymer of intrinsic microporosity
PLLA	Poly(L-lactic acid)
PMDA	Pyromellitic dianhydride
PMMA	Poly(methyl methacrylate)
PMP	Poly (4-methyl-1-pentyne)
PMPS	Polymethylphenylsiloxane
POZ	Polyoxazoline
PPEES	Poly-(1,4-phenylene ether-ether-sulfone)
PPO	Poly(2,6-dimethyl-1,4-phenylene oxide)
PSF	Polysulfone
PTMSP	Poly[1-(trimethylsilyl)-1-propyne]
PVAc	Poly(vinyl acetate)
SET-plan	European Strategic Energy Technology Plan
TED	Triethylenediamine
TPX	Polymethylpentene
XLPEO	Cross-linked polyethylene oxide
ZEP	Zero Emission Fossil Fuel Power Plants

ZIF	Zeolitic imidazolate framework

List of symbols

A	Membrane area	m^2
b_i	Affinity constant of specie i	Pa^{-1}
C_i	Concentration of component i	mol/m^3
C_i^{sat}	Langmuir capacity constant of specie i	mol/m^3
D_f	Diffusivity of specie i in the filler	m^2/s
D_i	Diffusion coefficient of component i	m^2/s
Đ$_i$	Maxwell-Stefan diffusivity of component i	m^2/s
D_i^{eff}	'Effective' diffusion coefficient of component i	m^2/s
D_i^H	Diffusivity of specie i in the Henry environment	m^2/s
D_i^L	Diffusivity of specie i in the Langmuir environment	m^2/s
$D_{Kn,i}$	Knudsen diffusion coefficient of component i	m^2/s
D_m	Diffusivity of specie i in the matrix	m^2/s
d_o	Pore diameter	m
$D_{self,i}$	Self-diffusivity of component i	m^2/s
F	Correction factor	-
f_i	Fugacity of component i	Pa
K	Interfacial equilibrium constant or partition coefficient	-
K_i	Langmuir isotherm adsorption constant	Pa^{-1}
K_i^H	Henry adsorption coefficient of specie i	$mol/(Pa \cdot m^3)$
L	Membrane thickness	m
M_i	Molecular weight of component i	kg/mol
N_c	Molar flux of a gas species in the continuous phase of a MMM	$mol/(m^2 \cdot s)$
N_d	Molar flux of a gas species in the dispersed phase of a MMM	$mol/(m^2 \cdot s)$
N_{eff}	Effective molar flux of a gas species in a MMM	$mol/(m^2 \cdot s)$
N_i	Molar flux of component i	$mol/(m^2 \cdot s)$
P_f	Permeability of specie i in the filler	$(mol \cdot m)/(m^2 \cdot s \cdot Pa)$
P_i	Permeability coefficient of component i	$(mol \cdot m)/(m^2 \cdot s \cdot Pa)$
p_i	Partial pressure of component i	Pa
P_m	Permeability of specie i in the matrix	$(mol \cdot m)/(m^2 \cdot s \cdot Pa)$
q_i	Adsorbed amount or loading of component i in a porous material	mol/kg
q_i^{sat}	Maxim adsorbed amount or loading of component i in a porous solid at saturation	mol/kg
R	Gas constant	$J/(mol \cdot K)$
R_a	Distance between Hansen parameters	$Pa^{0.5}$
S_{ij}	Ideal selectivity	-
T	Temperature	K
X_i	Mole fractions of component i	-
α_{ij}	Separation factor	-
Γ_{ii}	Thermodynamic correction factor	-

δ_D	Hansen solubility parameter to quantitatively describe the dispersion or London interaction between two phases	$Pa^{0.5}$
δ_H	Hansen solubility parameter to quantitatively describe the hydrogen bonds between two phases	$Pa^{0.5}$
δ_P	Hansen solubility parameter to quantitatively describe the polar interaction between two phases	$Pa^{0.5}$
Δpi	Partial pressure difference for component i across the membrane	Pa
E	Porosity	-
θ_i	Fractional coverage of the surface in a porous material	-
P	Density	kg/m^3
T	Tortuosity	-
ϕ_d	Volume fraction of the dispersed phase in a MMM	-

Table 2: Overview of the reported MOF-containing MMMs for gas separation in chronological order

M[4]		wt.% loading (best MMM performance)	Example (best performance)						Type of analysis	Operation conditions (optimal value)	
**MOF**	**Polymer**		***P CO_2*** (Barrer)	***CO_2/CH_4 selectivity*** (-)	***P CO_2*** (Barrer)	***CO_2/N_2 selectivity*** (-)	***P H_2*** (Barrer)	***H_2/CO_2 selectivity*** (-)		*T* (°C)	*ΔP* (bar)
Cu 4,4'-BPDC-TED	PAET	10-30 (30)	1.4-(0.7)	18.0-(3.2)	-	-	-	-	Single gas CO_2, O_2, N_2, CH_4	25	2
			-	-	-	-	-	-	Gas mixture CH_4/CO_2 (10:90)		
[$Cu_2(PF_6)(NO_3)(4,4'$-bpy$)_4]2PF_6·2H_2O$	PSF	2.5-5 (5)	-	-	-	-	-	-	Single gas He, H_2, O_2, N_2, CH_4	35	1
[Zn_2(1,4-bdc$)_2$(dabco)]·4DMF·0.5H_2O	PAI	30	46.7-(109)	49.7-(40.4)	46.7-(109)	28.3-(24.6)	79.2-(191)	1.7-(1.8)	Single gas	-	-
	6FDA-4MPD		1000-(3330)	23.0-(19.6)	1000-(3330)	21.4-(19.1)	743-(1890)	0.7-(0.6)			
	PDMS		2830-(4010)	3.4-(3.7)	2830-(4010)	10.5-(10.0)	673-(955)	0.2-(0.2)			
HKUST-1	PDMS	10-40(30, 10, 40)	2500-(2900)*	3.1-(3.6)*	2500-(3050)*	7.0-(8.9)*	550-(900)*	0.2-(0.4)*	Single gas H_2, CO_2, O_2, N_2, CH_4	-	-
	PSF	5-10 (5, 10)	6.5-(7.5)*	18-0-(21.5)*	6.5-(7.5)*	20.0-(25.0)*	9.8-(15.0)*	1.5-(1.9)*			
$Mn(HCOO)_2$	PSF	5-10 (10, 5)	6.5-(7.0)*	18.0-(9.5)*	6.5-(7.0)*	20.0-(25.5)*	9.5-(10.5)*	1.5-(1.6)*			
Cu-4,4'-BPY-HFS	Matrimid®	10-40 (20, 30)	7.3-(9.9)	34.7-(27.6)	7.3-(9.9)	33.1-(31.9)	17.5-(20.3)	2.4-(2.0)	Single gas H_2, CO_2, O_2, N_2, CH_4	35	2
		20	-	36.3-(20.5)	-	-	-	2.6-(2.6)	Gas mixture H_2/CO_2 (50:50, 75:25) CO_2/CH_4 (50:50, 10:90) CH_4/N_2 (94:6, 50:50)		
IRMOF-1	Matrimid®	20	10.0-(38.8)	28.2-(29.2)	-	-	33.1-(114.9)	3.3-(3.0)	Single gas H_2, CO_2, CH_4	50	7
	Ultem®	10, 20 (20)	2.0-(3.0)	30.3-(26.3)	-	-	11.2-(16.9)	5.7-(5.7)			
HKUST-1	Matrimid®	30	10.0-(22.1)	28.2-(29.8)	-	-	33.1-(66.9)	3.3-(3.0)			
MOF-5	Matrimid®	10-30 (30)	9.0-(20.2)	41.7-(44.7)	9.0-(20.2)	36.0-(38.8)	24.4-(53.8)	2.7-(2.7)	Single gas H_2, CO_2, O_2, N_2, CH_4	35	2

		30	-	38.0-(29.0)	-	-	-	2.3-(2.3)	Gas mixture<br>H_2/CO_2 (75:25, 50:50, 25:75)<br>CH_4/N_2 (94:6, 50:50, 25:75)<br>CO_2/CH_4 (10:90, 50:50, 25:75)		
ZIF-8	PPEES	10-30 (30)	6-(25)	-	6-(25)	-	-	-	-	30	1, 2, 3, 5, 7, 10
Cu 1,4-BDC	PVAc	15	2.4-(3.3)	34.9-(40.4)	2.4-(3.3)	32.1-(35.4)	-	-	Single gas He, CO_2, O_2, N_2, CH_4	35	4.5 (0.1 for CO_2)
ZIF-8	Matrimid®	20-60 (50)	9.5-(4.7)	39.7-(124.9)	9.5-(4.7)	30.6-(26.2)	28.9-(18.1)	3.0-(3.8)	Single gas H_2, CO_2, O_2, N_2, CH_4, C_3H_8	25	2.7
		50-60 (50, 60)	-	42.1-(89.2)	-	-	-	2.6-(7.0)	Gas mixture<br>H_2/CO_2 (50:50)<br>CO_2/CH_4 (10:90)		
HKUST-1	Matrimid®	10-30 (30)	10.0-(17.5)* (GPU)	18.0-(24.0)*	11.0-(18.5)* (GPU)	23.5-(24.5)*	-	-	Gas mixture<br>CO_2/CH_4 (10:90, 35:65, 75:25)<br>CO_2/N_2 (10:90, 35:65, 75:25)	35	10
HKUST-1	PMDA-ODA	3-6 (3, 6)	306.6-(227.2)	12.0-(7.0)*	306.6-(227.2)	8.0-(5.5)*	3066-(4445)	10.0-(27.8)	Single gas H_2, CO_2, O_2, N_2, CH_4	25	10
ZIF-90	Ultem®	15	1.4-(2.9)*	38-(39)*	-	-	-	-	Single gas CO_2, CH_4	35	4.5
	Matrimid®		7.5-(10.5)*	34-(35)*	-	-	-	-			
	6FDA-DAM		390-(720)	24-(37)	-	-	-	-	Gas mixture CO_2/CH_4 (50:50)	25	2
ZIF-20	PSF	8	-	-	-	-	-	-	Gas mixture O_2/N_2 (50:50)	35	2
NH_2-MIL-53(Al)	PSF	8, 16, 25, 40 (25)	2.0-(2.4)	45-(117)	-	-	-	-	Gas mixture CO_2/CH_4 (50:50)	-10, 35	1, 3, 5, 7, 10, 13
ZIF-7	PBI	10, 25, 50 (50)	-	-	-	-	3.7-(26.2)	8.7-(14.9)	Single gas H_2, CO_2	35	3.5
							75-(440)*	8.5-(7.2)*	Gas mixture H_2/CO_2 (50:50)	35, 60, 80, 120, 150, 180	7
ZIF-8 + S1C	PSF	16+0, 8+8 (16+0)	4.6-(12.1)	24.3-(19.8)	5.9-(12.3)	24.6-(19.5)	-	-	Gas mixture<br>CO_2/CH_4 (50:50)<br>CO_2/N_2 (50:50)	35	2

HKUST-1 + S1C		16+0, 8+8 (8+8)	4.6-(4.9)	24.3-(22.4)	5.9-(8.4)	24.6-(38.0)	-	-	O_2/N_2 (50:50)<br>H_2/CH_4 (50:50)		
HKUST-1	Matrimid®	10, 20, 30 (30)	10.0-(17.5)* (GPU)	18.5-(23.0)*	11.5-(19.5)* (GPU)	18.0-(23.5)*	-	-	Gas mixture<br>CO_2/CH_4 (10:90, 35:65, 75:25)<br>CO_2/N_2 (10:90, 35:65, 75:25)	35	10
ZIF-8			10.0-(22.5)* (GPU)	18.5-(19.5)*	11.5-(20.0)* (GPU)	18.0-(19.5)*	-	-			
MIL-53(Al)			10.0-(20.0)* (GPU)	18.5-(22.5)*	11.5-(20.0)* (GPU)	18.0-(23.0)*	-	-			
ZIF-8	PPEEs	10, 20, 30 (30)	5.4-(50.0)	22.9-(20.8)	5.4-(50.0)	30.1-(24.5)	6.7-(92.3)	1.3-(1.8)	Single gas<br>H_2, CO_2, O_2, N_2, CH_4, C_2H_4, C_2H_6	10, 20, 30, 40	1
ZIF-8	6FDA-DAM	16.4, 28.7, 48 (48)	-	-	-	-	-	-	Single gas C_3H_6, C_3H_8	35	2
									Gas mixture C_3H_6/C_3H_8 (50:50)		1.4, 2.8, 4.1, 5.5
MIL-101(Cr)	PSF	8, 16, 24	-	-	-	-	-	-	Single gas O_2, N_2	30	3
MOF-508a(Zn)		8	-	-	-	-	-	-			
MIL-53(Al)			-	-	-	-	-	-			
MIL-100(Fe)			-	-	-	-	-	-			
MIL-53(Al)	6FDA-ODA	25	14.5-(21.0)*	48.0-(44.0)*	-	-	-	-	Single gas CO_2, CH_4	35	10
			14.5-(21.0)*	42.0-(42.5)*	-	-	-	-	Gas mixture CO_2/CH_4 (50:50)		
NH_2-MIL-53(Al)		10, 15, 20, 25, 30, 32, 35 (32)	14.5-(14.7)*	48.0-(76.0)*	-	-	-	-	Single gas CO_2, CH_4		
			14.5-(14.7)*	42.0-(53.0)*	-	-	-	-	Gas mixture CO_2/CH_4 (50:50)		
ZIF-8	Ultem®	10, 13 (13)	-	-	14.0-(26.0) (GPU)	30.0-(36.0)	-	-	Single gas CO_2, N_2	25, 30, 35, 45	6.7
			-	-	(26.0)* (GPU)	(32.0)	-	-	Gas mixture CO_2/N_2 (20:80)	25, 35, 45	2.1, 2.8, 3.4
MIL-53(Al)	PMDA-ODA	5	0.30-(0.21) (GPU)	72.1-(50.5)	0.30-(0.21) (GPU)	34.8-(27.5)	0.35-(0.42) (GPU)	1.1-(2.0)	Single gas He, H_2, CO_2, N_2, CH_4	25	6

MOF-5			0.30-(0.27) (GPU)	72.1-(56.8)	0.30-(0.27) (GPU)	34.8-(14.1)	0.35-(0.24) (GPU)	1.1-(0.9)			
HKUST-1			0.30-(0.32) (GPU)	72.1-(73.6)	0.30-(0.32) (GPU)	34.8-(38.1)	0.35-(0.44) (GPU)	1.1-(1.3)			
ZIF-8	Matrimid®	10, 25 (25)	10.7-(23.2)	34-(39)	-	-	-	-	Single gas CO_2, CH_4	35	4.5
ZIF-8	Matrimid®	5, 10, 20, 30, 40 (20, 30)	8.1-(16.6)	35.2-(35.8)	8.1-(16.6)	22.4-(19.0)	32.7-(112.1)	4.0-(3.9)	Single gas H_2, CO_2, O_2, N_2, CH_4	22	4
UiO-66	6FDA-ODA	25	14.4-(50.4)	44.1-(46.1)	-	-	-	-	Single gas CO_2, CH_4	35	10
			-	41.7-(42.3)	-	-	-	-	Gas mixture CO_2/CH_4 (50:50)		
NH_2-UiO-66			14.4-(13.7)	44.1-(51.6)	-	-	-	-	Single gas CO_2, CH_4		
			-	41.7-(44.7)	-	-	-	-	Gas mixture CO_2/CH_4 (50:50)		
HKUST-1			14.4-(21.8)	44.1-(51.2)	-	-	-	-	Single gas CO_2, CH_4		
			-	41.7-(50.7)	-	-	-	-	Gas mixture CO_2/CH_4 (50:50)		
NH_2-HKUST-1			14.4-(26.6)	44.1-(59.6)	-	-	-	-	Single gas CO_2, CH_4		
			-	41.7-(52.4)	-	-	-	-	Gas mixture CO_2/CH_4 (50:50)		
UiO-67			14.4-(20.8)	44.1-(15.0)	-	-	-	-	Single gas CO_2, CH_4		
			-	41.7-(15.0)	-	-	-	-	Gas mixture CO_2/CH_4 (50:50)		
ZIF-8	PBI	18, 20, 29, 34, 59 (29)	-	-	-	-	3.7-(105.4)	8.6-(12.3)	Single gas H_2, CO_2	25	3.5
	PBI/ Matrimid®	10, 20, 33 (10)	-	-	-	-	2.1-(8.9) (GPU)	6.2-(9.5) (GPU)			
		10, 20, 33 (10)	-	-	-	-	(65.4) (GPU)	(12.3) (GPU)	Gas mixture H_2/CO_2 (50:50)	25, 80, 120, 150, 180	7
ZIF-8	6FDA-DAM:DABA 4:1	20	-	-	211.4-(553)	21.3-(19.3)	-	-	Single gas CO_2, N_2	30	1.4
ZIF-8	PBI	30, 60 (30)	-	-	-	-	4.1-(82.5)	8.9-(12.0)	Single gas H_2, CO_2	35	3.5
			-	-	-	-	(470)	(26.3)	Gas mixture H_2/CO_2 (50:50)	35, 60, 120, 180,	2

									$H_2/CO_2/CO$ (49.5:49.5:1)	230	
ZIF-7	Pebax®	8, 22, 34 (22)	72-(111)	14-(30)	72-(111)	34-(97)	-	-	Single gas CO_2, N_2, CH_4	20	6.5 (2.75 for CO_2)
ZIF-8	PIM-1	13.8, 24.2, 32.4, 39.0 (39.0)	4390-(6300)	14.2-(14.7)	4390-(6300)	24.4-(18.0)	1630-(6680)	0.4-(1.1)	Single gas He, H_2, CO_2, O_2, N_2, CH_4	20-22	1
HKUST-1	P84	20	-	-	-	-	-	-	Gas mixture C_2H_4/ C_2H_6 (80:20)	-	5, 10, 15
FeBTC			-	-	-	-	-	-			
MIL-53(Al)			-	-	-	-	-	-			
HKUST-1	P84	10, 20, 40 (20)	-	-	-	-	-	-	Gas mixture C_2H_4/ C_2H_6 (80:20)	-	5, 10, 15
ZIF-90	PBI	10, 25, 45 (45)	-	-	-	-	4.1-(24.5)	8.9-(25)	Single gas H_2, CO_2	35	3.5
		45	-	-	-	-	(226.9)	(13.3)	Gas mixture H_2/CO_2 (50:50)	35, 60, 80, 120, 180	7
HKUST-1	PPO	10, 20, 30, 40, 50 (40)	68.7-(115)*	16.4-(34)*	68.7-(115)*	16.0-(26)*	75.0-(119)*	1.1-(1.0)*	Single gas H_2, CO_2, N_2, CH_4	30	-
ZIF-8	6FDA-durene	33.3	468.5-(1552.9)	15.6-(11.0)	468.5-(1552.9)	13.4-(11.3)	518.5-(2136.6)	1.1-(1.4)	Single gas H_2, CO_2, O_2, N_2, CH_4	35	3.5
	6FDA-durene (cross-linked)		0.4-(23.7)	(16.9)	0.4-(23.7)	(11.9)	52.1-(283.5)	130.3-(12.0)			
NH_2-MIL-53(Al)	6FDA:DSDA-4MPD:4,4’-SDA 1:1	0, 5, 10, 15 (15)	57.9-(66.5)	35.1-(36.9)	-	-	90.1-(100)	1.6-(1.8)	Single gas H_2, CO_2, CH_4	35	3
	6FDA-4MPD:4,4’-SDA 1:1	10	134-(137)	30.2-(27.2)	-	-	169-(175)	1.3-(1.3)			

NH_2-MIL-101(Al)	6FDA:DSDA-4MPD:4,4'-SDA 1:1	0, 5, 10 (10)	57.9-(70.9)	35.1-(41.6)	-	-	90.1-(114)	1.6-(1.6)			
	6FDA-4MPD:4,4'-SDA 1:1	10	134-(151)	30.2-(29.6)	-	-	169-(191)	1.3-(1.3)			
NH_2-CAU-1	PMMA	5, 10, 15, 20, 25 (15)	-	-	-	-	5000-(11000)	3-(13)	Single gas H_2, CO_2	RT	3
			-	-	-	-	-	2-(10)*	Gas mixture H_2, CO_2 (-)		
MIL-68(Al)	PSF	4, 8 (8)	5.4-(4.7)	31.1-(36.5)	-	-	-	-	Gas mixture CO_2/CH_4 (50:50)	35	2
HKUST-1	PLLA	5	-	-	-	-	-	-	- CO_2, O_2	23	-
ZIF-8	6FDA-durene (400 °C)	20	541-(1090)	13.1-(13.0)	-	-	-	-	Single gas CO_2, CH_4, C_3H_6, C_3H_8	35	10 (3.5 for C_3H_6 and C_3H_8)
	6FDA-durene:DABA 9:1 (200 °C)	5, 10, 15, 20, 30, 40 (40)	256-(779)	19.5-(20.9)	-	-	-	-			
	6FDA-durene:DABA 7:3 (400 °C)	20	429-(698)	26.0-(25.8)	-	-	-	-			
	6FDA-durene:DABA 9:1 (400 °C)	20, 40 (20)	305-(728)	13.8-(19.6)	-	-	-	-	Gas mixture CO_2/CH_4 (50:50)	35	20
NH_2-MIL-53(Al)	Matrimid®	15	6.2-(9.2)	31.0-(2.1)	-	-	-	-	Single gas CO_2, CH_4	35	10
			-	28.5-(2.1)	-	-	-	-	Gas mixture CO_2/CH_4 (50:50)		
	Ultem®	15	1.5-(3.0)	39.5-(36.2)	-	-	-	-	Single gas CO_2, CH_4		
			-	31.6-(36.1)	-	-	-	-	Gas mixture CO_2/CH_4 (50:50)		
	6FDA-ODA:DAM 1:1	15, 20, 22 (10)	54.1-(51.2)	23.5-(34.1)	-	-	-	-	Single gas CO_2, CH_4		
			-	23.6-(31.8)	-	-	-	-	Gas mixture CO_2/CH_4 (50:50)		

	6FDA-ODA:DAM 1:4	10, 15, 20 (15)	130.0-(113)	23.2-(28.2)	-	-	-	-	Single gas CO_2, CH_4		
			-	23.6-(28.5)	-	-	-	-	Gas mixture CO_2/CH_4 (50:50)		
	6FDA-ODA:DAM 1:1 (APTMDS)	15, 20, 25, 30, 32, 35 (30)	32.2-(58.5)	18.9-(36.6)	-	-	-	-	Single gas CO_2, CH_4		
			-	20.2-(33.9)	-	-	-	-	Gas mixture<br>CO_2/CH_4 (10:90)<br>CO_2/CH_4 (35:65)<br>CO_2/CH_4 (50:50)<br>CO_2/CH_4 (60:40)<br>CO_2/CH_4 (80:20)<br>CO_2/CH_4 (85:15)	30, 35, 45, 60, 75	3.4-41 (10)
MIL-53	Matrimid®	15	6.2-(6.7)	31.0-(9.4)	-	-	-	-	Single gas CO_2, CH_4	35	10
			-	28.5-(8.5)	-	-	-	-	Gas mixture CO_2/CH_4 (50:50)		
	Ultem®	15	1.5-(1.8)	39.5-(43.1)	-	-	-	-	Single gas CO_2, CH_4		
			-	31.6-(42.8)	-	-	-	-	Gas mixture CO_2/CH_4 (50:50)		
	6FDA-ODA:DAM 1:1	20	54.1-(61.5)	23.5-(12.5)	-	-	-	-	Single gas CO_2, CH_4		
			-	23.6-(13.0)	-	-	-	-	Gas mixture CO_2/CH_4 (50:50)		
	6FDA-ODA:DAM 1:4	25	130.0-(123)	23.2-(18.1)	-	-	-	-	Single gas CO_2, CH_4		
			-	23.6-(19.1)					Gas mixture CO_2/CH_4 (50:50)		
	6FDA-ODA:DAM 1:1 (APTMDS)	25	32.2-(76.4)	18.9-(8.9)					Single gas CO_2, CH_4		
			-	20.2-(8.8)	-	-	-	-	Gas mixture CO_2/CH_4 (50:50)		
TKL-107	Matrimid®	5, 10, 20, 30 (20)	7-(17)*	36-(64.6)	-	-	-	-	Single gas CO_2, CH_4	25	2
			6-(15)*	24-(50.3)	-	-	-	-	Gas mixture CO_2/CH_4 (20:80)		
CPO-27(Mg)	XLPEO	10	-	-	380-(250)	22-(25)	-	-	Single gas CO_2, N_2	25	2
	6FDA-TMPDA	10	-	-	650-(850)	14-(23)	-	-			
	PDMS	20	-	-	3100-(2100)	9.5-(12)	-	-			
Silica-(ZIF-8) core-shell	PSF	8, 12, 16, 20, 32 (32)	11.8-(73.1)*	10.2-(5.5)*	-	-	35.0-(224.1)*	3.4-(3.9)*	Gas mixture<br>H_2/CO_2 (50:50)<br>CO_2/CH_4 (10:90, 50:50, 90:10)	35, 60, 90,120, 150	2

									35, 60, 90, 120	
ZIF-8	Pebax®	5, 10, 15, 20, 25, 30, 35 (35)	351-(1287)	8.3-(9.0)	351-(1287)	33.8-(32.3)	-	-	Single gas CO_2, O_2, N_2, CH_4	RT	2, 6
		5, 10, 15, 20, 25, 30, 35, 40, 50 (25)	-	-	200-(900)*	66-(53)*	-	-	Gas mixture CO_2/N_2 (10:90)	25	1.5
MIL-53	Matrimid®	5, 10, 15, 20 (15)	6.4-(12.4)	28.2-(51.8)	-	-	-	-	Single gas CO_2, CH_4	35	3
ZIF-8	PSF	8	-	-	-	-	-	-	Gas mixture H_2/CH_4 (50:50) O_2/ N_2 (50:50)	35	2
NH_2-MIL-53(Al)			-	-	-	-	-	-			
$[Cd_26FDA(H_2O)]_25$ H_2O	6FDA-ODA	10	20.6-(37.8)	33.1-(44.8)	20.6-(37.8)	26.4-(35.1)	-	-	Single gas CO_2, N_2, CH_4	25	2
MIL-53(Al)	PMP	5, 10, 15, 20, 25, 30, 35, 40 (30)	-	-	-	-	100-(365)*	0.11-(0.04)	Single gas H_2, CO_2	30	2, 4, 6, 8
ZIF-8	6FDA-Durene	3, 5, 7, 10, 15, 20, 30 (30)	1468.3-(2185.5)	22.6-(17.1)	1468.3-(2185.5)	25.4-(17.0)	-	-	Single gas CO_2, O_2, N_2, CH_4	RT	2, 6
			-	-	-	-	-	-	Gas mixture CO_2/N_2 (10:90)	25	1.5
NH_2-MIL-53(Al) + MSSs	PSF	16+0, 12+4, 8+8, 4+12 (4+12)	-	-	-	-	-	-	Gas mixture O_2/N_2 (50:50) H_2/CH_4 (50:50)	35	2
NH_2-MIL-53(Al) + MSSs	Matrimid®	8+8, 4+12 (4+12)	-	-	-	-	-	-			
NH_2-MIL-53(Al)	Matrimid®	15, 20, 25 (25)	4.8-(3.9)	100-(107)	-	-	-	-	Gas mixture CO_2/CH_4 (50:50)	0, 25, 35	3, 5, 9, 12
	PSF	15, 20, 25 (25)	5.2-(5.4)	23.0-(27.5)	-	-	-	-		35	3
NH_2-MIL-101(Al)	Matrimid®	8, 15, 25 (25)	4.8-(3.0)	100-(98)	-	-	-	-		0, 25, 35	3, 5, 9, 12
	PSF	8, 15, 25 (25)	5.2-(8.4)	23.0-(28.5)	-	-	-	-		35	3
ZIF-8	6FDA-DAM	17, 30 (17)	-	-	-	-	-	-	Single gas O_2, N_2	35	2
									Gas mixture C_3H_6, C_3H_8 (50:50)		1.4
ZIF-8	Matrimid®	15	9-(26)*	34.5-(35)*	-	-	-	-	Single gas CO_2, CH_4	35	3.45
ZIF-7-8-(20)			9-(20)*	34.5-(35.5)*	-	-	-	-			
ZIF-8-ambz-(15)			9-(12)*	34.5-(36)*	-	-	-	-			

ZIF-8-ambz-(30)			9-(11)*	34.5-(38.5)*	-	-	-	-			
ZIF-7-8-(20)			8-(19)*	43-(41)*	-	-	-	-	Gas mixture CO_2/CH_4 (50:50)	-	6.9, 13.8, 27.6, 41.4
ZIF-8-ambz-(15)			8-(14)*	43-(40)*	-	-	-	-			
ZIF-8-ambz-(30)			8-(11)*	43-(42.5)*	-	-	-	-			
FeBTC	Matrimid®	10, 20, 30	14-(14)*	55-(35)*	-	-	-	-	Single gas CO_2, CH_4	35	2-40 (40)
			14-(8.2)*	22-(28)*	-	-	-	-	Gas mixture CO_2/CH_4 (50:50)	-	5
ZIF-8	PBI-BuI	10, 20, 30 (30)	2.3-(5.2)	57.0-(43.6)	2.3-(5.2)	26.8-(16.0)	6.2-(22.1)	2.7-(4.2)	Single gas He, H_2, CO_2, N_2, CH_4	35	20
	DMPBI-BuI	10, 20, 30 (30)	3.8-(53.9)	47.2-(15.7)	3.8-(53.9)	21.7-(11.3)	12.8-(127.5)	3.4-(2.4)			
	DBzPBI-BuI	10, 20 (20)	25.8-(89.8)	15.9-(11.6)	25.8-(89.8)	12.9-(14.3)	61.4-(180.3)	2.4-(2.0)			
NH_2-MIL-53(Al)	PMP	5, 10, 15, 20, 25, 30, 35, 40 (30)	96.5-(358.2)	8.8-(24.4)	-	-	-	-	Single gas CO_2, CH_4	30	2, 4, 6, 8
			80.1-(339.5)	8.1-(22.9)	-	-	-	-	Gas mixture CO_2/CH_4 (10:90)		
MIL-53(Al)-ht	Matrimid®	33.3, 37.5 (37.5)	8.4- (51)	39.4- (47.0)	8.4- (51)	33.6- (28.3)	25.7-(103)	3.1- (2.0)	Single gas H_2, CO_2, O_2, N_2, CH_4	35	2
MIL-53(Al)-as		37.5	8.4- (40)	39.4- (90.1)	8.4- (40)	33.6- (95.2)	25.7-(66.0)	3.1- (1.7)			
c-MOF-5	PEI	5, 15, 25 (25)	1.7- (5.4)	18.7- (23.4)	1.7- (5.4)	16.8- (28.4)	10.1-(28.3)	6.0- (5.3)	Single gas H_2, CO_2, N_2, CH_4	25	6
HKUST-1	Ultem®	10, 20, 30, 35, 40 (35)	1.1- (4.1)	36.8- (34.0)	1.1-(4.1)*	28.0-(28.0)*	-	-	Single gas CO_2, O_2, N_2, CH_4	35	3.5
HKUST-1	ODPA-DAM (annealed 200 °C 24 h)	10, 15, 20, 30, 40, 50 (40)	47.7-(260.7)	29-(28)*	-	-	-	-	Single gas CO_2, O_2, N_2, CH_4	35	2
	Matrimid® (annealed 200 °C 24 h)	20	7.6-(24.8)	37.5-(37.8)	-	-	-	-			
ZIF-8	ODPA-DAM (annealed 200 °C 24 h)		47.7-(134)*	29-(26)*	-	-	-	-			

ZIF-71	6FDA-durene	10, 20, 30 (20)	959-(4006)	16.4-(12.8)	959-(4006)	14.7-(12.9)	756-(2310)	0.8-(0.6)	Single gas H_2, CO_2, O_2, N_2, CH_4, C_2H_4, C_2H_6, C_3H_6, C_3H_8	35	3.5 (2 for C_2H_4, C_2H_6, C_3H_6 and C_3H_8)
		10, 20, 30 (20)	917-(3435)	21.8-(16.0)	-	-	-	-	Gas mixture CO_2/CH_4 (50:50)	35	7
$[Cu_2(Glu)_2(\mu\text{-}bpa)]\cdot(CH_3CN)$	POZ	5, 10, 15, 20 (15)	-	-	28-(11.6)*	1-(55)*	-	-	Single gas CO_2, N_2	-	3.1
$[Cu_2(Glu)_2(\mu\text{-}bpp)]\cdot(C_3H_6O)$			-	-	28-(16.0)*	1-(7)*	-	-			0.4
MIL-53(Al)	Matrimid®	10, 20, 30 (30)	14-(24)*	55-(66)*	-	-	-	-	Single gas CO_2, CH_4	35	2.5, 5, 7.5, 10, 12.5, 15, 20, 25, 30, 40
ZIF-8			14-(24)*	55-(72)*	-	-	-	-			
HKUST-1			14-(18)*	55-(52)*	-	-	-	-			
MIL-53(Al)			9-(18)*	5-(40)*	-	-	-	-	Gas mixture CO_2/CH_4 (50:50)		2.5, 5, 7.5, 10, 15, 20
ZIF-8			9-(20)*	5-(37)*	-	-	-	-			
HKUST-1			9-(14)*	5-(46)*	-	-	-	-			
ZIF-11	PSF	4.7	-	-	-	-	-	-	-	-	-
	PES	4.7	-	-	-	-	-	-			
	PBI	16.1, 29.7, 39.5 (39.5)	-	-	-	-	17.2-(464.7)	5.0-(3.6)	Single gas H_2/CO_2	RT	-
b-Cu 1,4-BDC	Matrimid®	8	5.8-(5.2)	59.8-(45)	-	-	-	-	Gas mixture CO_2/CH_4 (50:50)	25	3, 4.5, 6, 7.5
nc-Cu 1,4-BDC		8	5.8-(5.0)	59.8-(49.4)	-	-	-	-			
ns-Cu 1,4-BDC		2, 4, 8 (8)	5.9-(2.8)	47.7-(88.2)	-	-	-	-			3, 4.5, 6, 7.5
ns-Cu 2,6-NDC		8	5.8-(6.3)	59.8-(43.5)	-	-	-	-			3
ZIF-8 100 nm	PSF	5	25.7-(15.6) (GPU)	19.4-(28.5)	-	-	-	-	Single gas CO_2, CH_4	27	4
ZIF-8 300 nm			25.7-(25.9) (GPU)	19.4-(5.8)	-	-	-	-			

ZIF-8 500 nm			25.7-(28.1) (GPU)	19.4-(5.8)	-	-	-	-			
ZIF-8	PDMS	2.5, 5, 10, 15, 20	-	-	-	-	-	-	Single gas C_3H_8, N_2	-	1.5
			-	-	-	-	-	-	Gas mixture C_3H_8/N_2 (10:90, 20:80, 30:70, 40:60)	20, 27, 35, 45, 55	0.5, 1.5, 2.5, 3.5, 4.5

Results with * are calculated from graphs.
